# Theta oscillations are an organizational unit of odor processing in the olfactory bulb

**DOI:** 10.1126/sciadv.aee1002

**Published:** 2026-07-03

**Authors:** Andrew Sheriff, Mahmoud Omidbeigi, Gregory Lane, Qiaohan Yang, Guangyu Zhou, Adam Dede, Naelly Arriaga, Vivek Sagar, Ania M. Holubecki, Rodrigo M. Braga, Leslie M. Kay, Bruce K. Tan, Christina Zelano

**Affiliations:** ^1^ Department of Neurology, Northwestern University Feinberg School of Medicine, Chicago, IL, USA.; ^2^ Department of Psychology, The University of Chicago, Chicago, IL, USA.; ^3^ Institute for Mind and Biology, The University of Chicago, Chicago, IL, USA.; ^4^ Department of Otolaryngology—Head and Neck Surgery, Northwestern University Feinberg School of Medicine, Chicago, IL, USA.

## Abstract

When sampling odors, humans typically take a single long sniff, unlike most other mammals who sample odors through bouts of rapid repetitive sniffing. Decades of work has established that rapid sniffing rhythms underlie the organizational principles of odor coding, with sniff speed clocking odor responses in the olfactory bulb. In the absence of rapid sniffing, how are odor responses organized in the human olfactory system? Because most mammals sniff at rates centered around the theta frequency, we hypothesized that the olfactory bulb exploits a theta-range neural oscillation to set the pace of odor coding in the system. Here, we used high-precision neural recordings from the human olfactory bulb to show that initiation of a sniff elicits and temporally aligns theta oscillations in the bulb and that sniff-induced theta oscillations organize the timing and amplitude of odor responses. These findings suggest that, despite the lack of rapid sniffing bouts in humans, the system has preserved a similarly timed unit of olfactory processing.

## Teaser

The olfactory bulb organizes odor responses according to theta rhythms even in the absence of theta-range sniffing.

## INTRODUCTION

Across the brain, neuronal activity is timed to sensory sampling behavior, allowing for optimization of stimulus responses (*1*–*3*). In the mammalian olfactory system, sniffing controls the timing of odor delivery to olfactory receptors inside the nasal cavity, and each sniff evokes a precise sequence of activity across olfactory neurons in the brain (*4*, *5*). Numerous studies suggest that, in rodent olfaction, neuronal responses are tuned to the sniff rhythm (*4*–*20*): Odor information is encoded both in the number of spikes fired per sniff cycle and in the timing of those spikes relative to sniff phase (*4*–*11*). Furthermore, odors evoke olfactory bulb activity that is precisely sniff-locked, with single-cell firing times tiling the duration of the sniff cycle (*4*). The timing of these responses is tightly locked to sniff phase, with exceptional precision across trials (*4*, *21*, *22*). Thus, in rodents, the sniff cycle is thought to be the fundamental temporal unit of olfaction (*23*) constraining the time window of relevant neural processing (*5*, *22*), at least partially (*4*, *7*).

How do these findings translate to humans, who typically take single long (3 to 5 s) sniffs when encountering odors? Despite markedly slower sniffing behavior, the human olfactory system achieves temporal precision that is comparable to rodents (*24*), indicating that the sniff rhythm itself cannot be the only temporal unit of neural processing in human olfaction. This presents two possibilities: that a fundamental mechanism of odor processing is not conserved across mammalian species in an otherwise highly conserved system or that there exists a rhythm faster than the human sniff that organizes the timing of odor processing. Because animal and human olfactory systems and their physiological signatures are generally highly conserved within mammals and recur via independent evolution across several phyla (*25*–*27*), the latter possibility appears more likely.

A strong candidate for a conserved rhythm is the olfactory bulb theta oscillation, which overlaps in frequency with the rodent sniff rhythm (*17*, *28*, *29*). Rodent olfactory bulb theta activity has been well characterized by decades of local field potential (LFP) work (*17*, *22*, *30*–*32*), has been hypothesized to gate sensory windows in olfaction (*33*), and has been shown to couple with gamma power in a learning-dependent manner (*34*). Thus, theta rhythms are well positioned to contribute to temporal constraints of olfactory processing in the rodent olfactory bulb. However, the field of olfaction has largely focused on the sniff, rather than the theta oscillation, as an organizing unit. Furthermore, the strong coherence between olfactory bulb theta and sniffing makes these two rhythms difficult to disentangle in rodents. In humans, sniffing rhythms are much slower and therefore diverge from theta rates. Thus, using humans as an animal model (*35*) presents an opportunity to separately examine the contribution of sniffing rhythms and theta rhythms to the organization of olfactory processing. It is currently unknown whether the human olfactory bulb exhibits theta oscillations and, if so, whether they play a functional role in organizing odor responses.

To address this gap, we combined nasal airflow recordings with high-precision electrophysiological recordings from the olfactory bulb in healthy human volunteers, including single-trial quantification of sniff-related neural oscillations, in air and odor conditions, to detect and analyze olfactory bulb theta and its relationship to respiration and odor processing. We hypothesized that single human sniffs align theta oscillations in the olfactory bulb, which, in turn, organize the timing of gamma oscillations, which are linked to odor processing.

## RESULTS

### High-precision recordings from the human olfactory bulb

**Fig. 1.**
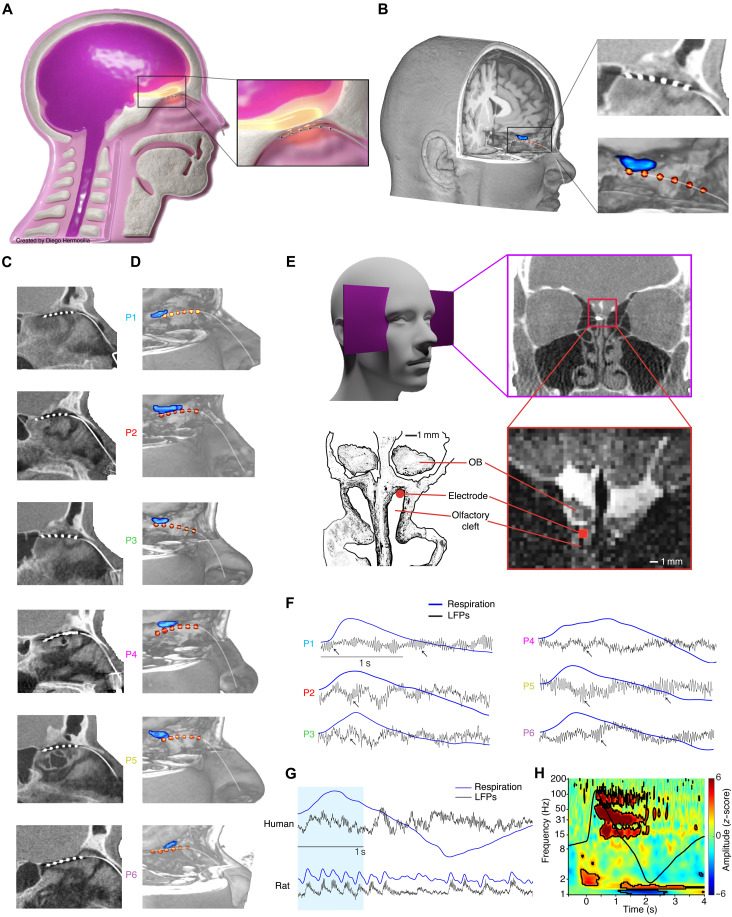
High-precision neural recordings from the human olfactory bulb. (**A**) Schematic of the electrode at the top of the nasal cavity, below the cribriform plate. Olfactory bulb: yellow. (**B**) Left: 3D rendering of coregistered CT, T1-weighted, and T2-weighted MRI from a representative participant (P3). Olfactory bulb mask from T2-weighted MRI (blue) and electrode mask from CT (red/yellow) are shown. Right: Zoomed sagittal CT image (top; white dots are electrode contacts) and 3D rendering (bottom; red/yellow dots are the electrode mask, and blue is the olfactory bulb mask) of electrode placement below the cribriform plate. (**C**) Sagittal CT (*N* = 6) following electrode placement by author B.K.T. Electrode contacts are white dots. (**D**) 3D renderings of coregistered CT, T1-weighted, and T2-weighted MRI (*N* = 6) showing the electrode location (red/yellow) relative to bulb (blue) following placement. (**E**) Top left: Schematic of the coronal slice used. Bottom left: Schematic of the electrode placement in the coronal plane [adapted from Low *et al.* (*120*)]. Red dot represents electrode contact. Top right: Coronal CT showing the electrode placement in a representative participant. Electrode contact is the white dot at the top of the olfactory cleft. Bottom right: Coronal T2-weighted MRI showing the electrode placement in a representative participant. The red dot is electrode contact (from coregistered CT). (**F**) Examples of raw (unfiltered) olfactory bulb LFP recordings (black) and respiration (blue) from six participants, showing visible gamma (small black arrows) and theta oscillations. (**G**) Olfactory bulb LFP recordings (black) from a human (top) and a rat (bottom) (*89*) during odor sampling, with corresponding respiratory signals (blue). Similar frequency of theta and gamma oscillations are visible following sniffs across species (light blue panel), despite differences in sniffing behavior. (**H**) Amplitude spectrogram aligned to sniff onsets (time = 0) from a representative participant (P1), showing significant increases in theta, beta, and gamma frequency bands (FDR-corrected significance threshold, *P* = 0.0049, *N* = 22 trials, permutation test against pre-sniff baseline).

Testing these hypotheses required measuring human olfactory bulb LFPs at a high spatial and temporal precision, allowing for single-trial assessment of both theta and gamma oscillations. To accomplish this, we recorded electrophysiological signals on a multicontact electrode (Ad-Tech Macro-Micro depth electrode) placed intranasally along the top of the nasal cavity below the cribriform plate, at a distance of ∼1 mm from the ventral surface of the olfactory bulb (see Materials and Methods; Fig. 1, A to E). Participants included six healthy volunteers (Fig. 1, C and D). We obtained robust recordings of odor-driven LFPs, with both theta (2 to 8 Hz) and gamma (>30 Hz) oscillations visible in raw (unfiltered) single trials (Fig. 1F), that were qualitatively similar to rodent olfactory bulb oscillations (Fig. 1G) and to prior human intracranial work (*36*, *37*). Consistent with rodent olfactory bulb recordings (*30*), time-frequency analyses showed statistically significant odor-induced responses in the theta, beta, and gamma ranges (Fig. 1H).

We then conducted a series of control experiments to confirm that we were recording from the olfactory bulb. First, to determine whether odor responses were diminished in the absence of an olfactory bulb, we compared recordings from a normosmic participant with those from a congenitally anosmic participant who lacked olfactory bulbs but had otherwise normal cognition. Second, to further control for the possibility that odor responses were due to cognitive processing in the brain rather than odor responses in the olfactory bulb, we conducted a tightly controlled cue-matching experiment that included comparison of carefully balanced olfactory and visual conditions. Third, because olfactory bulb circuits generate strong gamma oscillations in response to odor (*36*–*46*), we compared the strength of odor-evoked gamma oscillations to those in response to air. Fourth, to confirm the spatial specificity of gamma oscillations to the olfactory bulb, we quantified power on electrode contacts at varying distances from the OB. Fifth, we reasoned that if volume-conducted signals from the brain were driving our results, then direct intracranial recordings from the ventral surface of the orbitofrontal cortex, above the olfactory bulb, should show similar effects to those we observed intranasally; therefore, we analyzed existing data collected in our lab from participants performing a similar olfactory cue task with intracranial electrodes surgically implanted in ventral orbitofrontal areas to determine whether odor-evoked activity patterns from those areas looked similar to our recordings.

**Fig. 2.**
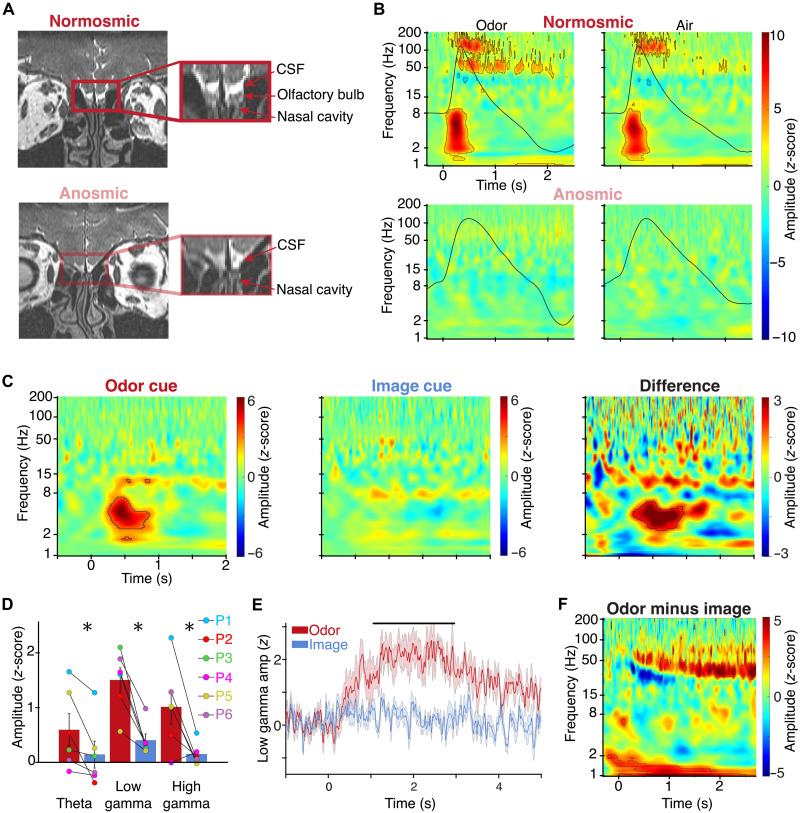
Controls: Comparison to anosmic and other sensory modalities. (**A**) Top: Coronal T2-weighted MRI from a representative normosmic participant (P1) showing normal olfactory bulbs. Bottom: Same from a congenital anosmic showing lack of olfactory bulbs. Cerebrospinal fluid (CSF) is white. (**B**) Amplitude spectrograms aligned to the onset of sniffs containing odor (left) and air (right), for a representative normosmic (P1; top) and congenital anosmic patient (bottom). The normosmic participant exhibited statistically significant odor responses, outlined in black (FDR-corrected threshold at *P* = 0.0096, *N* = 20 trials, permutation test against pre-sniff baseline), whereas the anosmic patient exhibited no statistically significant responses to odor. (**C**) Amplitude spectrograms aligned to cue onset in the odor condition (left), in the visual condition (middle), and in the difference between conditions (right). Statistical significance is outlined in black. Odor cues elicited significant theta oscillations (FDR-corrected significance threshold at *P* = 0.0016, *N* = 240 trials, permutation test against pre-cue baseline), whereas image cues elicited no significant responses. A direct comparison shows that theta oscillations were significantly greater in response to odor versus image cues (*P* < 0.05, *N* = 240 trials, permutation test of amplitude differences, cluster corrected). (**D**) Significant increases in theta (*T*_5_ = 2.70, *P* = 0.04), low gamma (*T*_5_ = 5.30, *P* = 0.003), and high gamma (*T*_5_ = 3.11, *P* = 0.03) for odors (red) compared to images (blue) (*N* = 6 participants, two-sided paired *t* tests, *P < 0.05). (**E**) Trial-averaged time series of low gamma (30 to 60 Hz) amplitude for odor (red) and image (blue) stimuli. Statistically significant differences indicated by a black bar (*N* = 6 participants, cluster-corrected *P* < 0.05, participant-level cluster-based permutation test). (**F**) Theta and gamma amplitudes are increased in response to odors compared to images (*P* < 0.05, *N* = 240 trials, permutation test of amplitude differences, cluster corrected).

In our first control experiment, we compared odor responses from a normosmic participant (*N* = 1) with responses from a congenitally anosmic participant [*N* = 1, University of Pennsylvania Smell Identification Test (UPSIT) score of 8], who had no discernable olfactory bulbs on a high-resolution T2-weighted magnetic resonance imaging (MRI) (Fig. 2A). In contrast to statistically significant odor responses in the normosmic [false discovery rate (FDR)–corrected threshold at *P* = 0.0096, permutation test against pre-sniff baseline], we found no significant responses to sniffs of odor or clean air in the congenitally anosmic participant (Fig. 2B). This suggests that the odor-induced responses observed in normosmic participants originated in the olfactory bulb rather than elsewhere in the brain and were induced by sniffing and the presence of odor rather than other cognitive aspects of the task.

In our second control experiment, we further investigated the possibility that our observed responses were due to cognitive processing originating elsewhere in the brain. We reasoned that if our signals originated in the olfactory bulb, they would be enhanced in an olfactory task compared to an identical visual task. We conducted a tightly controlled cue-matching experiment that was carefully balanced across olfactory and visual modalities. For both conditions, each trial began with a visual cue consisting of a written and spoken word (e.g., banana), followed by a delay after which an odor (e.g., the odor of banana) was presented in the olfactory version and a picture (e.g., a picture of a banana) was presented in the visual version (fig. S1). Participants then indicated whether the stimulus matched the cue. Across conditions, an identical cue therefore took on two different meanings: in one case indicating that an odor would arrive soon and in the other case indicating that a picture would arrive soon. Critically, the cues were otherwise identical, and no odor or picture was presented during the cues. If our signals originated in the olfactory bulb, which receives massive top-down projections and responds to olfactory cues (*47*–*52*), we would expect to see enhanced responses to the cues as well as to their corresponding stimuli presented during the olfactory version. We found that odor cues elicited significant theta oscillations (FDR-corrected significance threshold at *P* = 0.0016, permutation test against pre-cue baseline) that were significantly greater than responses to visual cues (*P* < 0.05, permutation test of amplitude differences, cluster corrected) (Fig. 2C). Furthermore, we found that odor stimuli elicited significantly greater amplitude of theta (*T*_5_ = 2.70, *P* = 0.04), low gamma (*T*_5_ = 5.30, *P* = 0.003), and high gamma (*T*_5_ = 3.11, *P* = 0.03) oscillations than visual stimuli, suggesting that these responses were from the olfactory bulb (Fig. 2D and fig. S2). Low gamma amplitude was significantly increased after odor compared to image onset (*P* < 0.05, *N* = 40 trials, permutation test of amplitude differences, cluster corrected) (Fig. 2E). A direct comparison of amplitude in response to odors versus images showed a significant increase in theta and gamma oscillations for odors (*P* < 0.05, permutation test of amplitude differences, cluster corrected) (Fig. 2F).

**Fig. 3.**
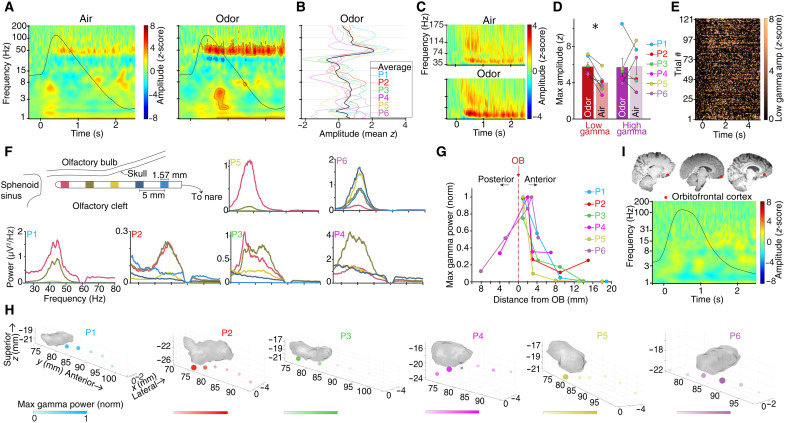
Controls: Presence of gamma and spatial specificity of responses to the olfactory bulb. (**A**) Amplitude spectrograms of sniff-onset-aligned responses to air (left) and odor (right) ( FDR-corrected significance threshold at *P* = 0.0036, *N* = 122 trials, permutation test) Black outline indicates significance. (**B**) Amplitude averaged over the first 1 s following sniff onset at each frequency, for each participant (colored lines), and averaged across participants (black line). (**C**) Amplitude spectrograms for air (top) and odor (bottom), with significant differences in low gamma (fig. S3, *P* < 0.05, *N* = 122 trials, cluster-based permutation test). (**D**) Bar plots showing the average maximum amplitude of low and high gamma across participants (colored dots), for odor and air conditions. The asterisk (*) denotes statistical significance (*N* = 40 trials, low gamma: *T*_5_ = 4.89, *P* = 0.0045, two-sided paired *t* test; high gamma: *T*_5_ = −0.076, *P* = 0.94). (**E**) Single-trial raster plot of low gamma amplitude following sniff onset (time = 0) for 122 trials of odor. (**F**) Schematic of the electrode in the olfactory cleft with colors of each contact corresponding to contact-wise power spectra for each participant. Power spectra were averaged over sniffs of odor during odor cue task (*N* = 40 trials). (**G**) Maximum gamma power of each electrode contact plotted against the distance from the ventral surface of the olfactory bulb for each participant. (**H**) Olfactory bulb and electrode contact locations visualized in 3D space. Gamma power is represented by the size and color saturation of each contact. Gray “x”: damaged contact. (**I**) Intracranial EEG data from three participants from a prior study (*55*) who performed an olfactory cue task with electrodes (red dots) implanted in the medial inferior orbitofrontal cortex above the olfactory bulb (top; T1-weighted MRIs). Amplitude spectrogram of sniff-onset-aligned responses to odor (bottom; *N* = 3, FDR-corrected permutation test against pre-sniff baseline; no areas surpassed the statistical threshold).

In our third control experiment, based on findings that rodent olfactory bulb circuits generate strong gamma oscillations in response to odor (*38*–*44*), we looked for evidence in our recordings of stronger gamma oscillations in response to odor, relative to air. We analyzed neural oscillations during sniffs of odor versus clean air during an odor detection task, and found significantly increased amplitude of gamma oscillations (FDR-corrected significance threshold at *P* = 0.0036, *N* = 122 trials, permutation test) under the odor condition (Fig. 3, A to D). Furthermore, similar to rodents, the olfactory bulb exhibited activity in distinct gamma bands (*53*, *54*): a high gamma (60 to 150 Hz) and a low gamma (30 to 60 Hz) (Fig. 3, C and D). The tight frequency range of the low gamma band was highly consistent across individual participants (Fig. 3B) and was significantly stronger for odor relative to air (*P* < 0.05, cluster-corrected, permutation test) (fig. S3). High and low gamma exhibited different response profiles across conditions. High gamma responses were similar for odor and air (*T*_5_ = −0.076, *P* = 0.94), whereas low gamma showed consistently stronger responses to odor than to air in every participant (*T*_5_ = 4.89, *P* = 0.0045) (Fig. 3D). Low gamma odor-induced responses were robust and evident at the single-trial level (Fig. 3E).

In our fourth control experiment, we reasoned that if these gamma oscillations were originating in the olfactory bulb, then their magnitude should decrease with distance from the olfactory bulb. We therefore quantified the strength of gamma oscillations recorded at each contact along the electrode, comprising an ∼25-mm recording area at varying distances from the olfactory bulb, for every participant (Fig. 3F). We plotted these values against the Euclidian distance from each participant’s olfactory bulb and found a steep drop in gamma power as distance from the olfactory bulb increased, in every participant (Fig. 3G). In a second analysis, we generated three-dimensional (3D) renderings of each participant’s olfactory bulb (obtained from high-resolution T2-weighted MRIs) and aligned these to the computed tomography (CT) scan acquired after electrode placement, so that the electrode contacts could be viewed relative to bulb proximity. We then overlaid the magnitude of gamma power onto the electrode contacts as a visual map, such that color (stronger color indicating stronger gamma power) and size (larger size indicating stronger gamma power) corresponded to gamma strength. This analysis provided a transparent view of gamma strength relative to olfactory bulb location and showed that gamma power drops off with distance from the bulb and, in particular, in both the anterior and posterior directions in two participants whose electrodes spanned both directions (P4 and P6; Fig. 3H). These analyses combine to suggest spatial localization of the effects to the olfactory bulb.

In our fifth control experiment, to further examine potential contributions to our results from volume-conducted signals originating elsewhere in the brain, we analyzed previously collected intracranial electroencephalography (iEEG) data (*55*) from three participants who had electrodes implanted in the orbitofrontal cortex in the medial inferior region above the olfactory bulb and who performed an olfactory cue task similar to the one in the present study. We reasoned that if the signals we recorded intranasally contained significant contributions from the orbitofrontal cortex, then we should see similar effects in intracranially positioned orbitofrontal electrodes. On the other hand, if our signals were originating from the bulb, then responses during the olfactory cue task should look different (or nonexistent) in the orbitofrontal cortex. The results of this analysis revealed no significant responses to odor in the medial inferior orbitofrontal cortex (Fig. 3I).

Together, these results suggest that our recordings provide high-precision signals originating in the human olfactory bulb.

Following these control analyses, we identified the electrode contact closest to the olfactory bulb for each participant (Fig. 3H) and used signals recorded from this contact for all remaining analyses. For all analyses, theta band was defined as 2 to 8 Hz, in line with prior human intracranial work (*56*, *57*), and gamma band was defined as 30 to 150 Hz.

### Intentional sniffs enhance and align theta oscillations in the olfactory bulb

**Fig. 4.**
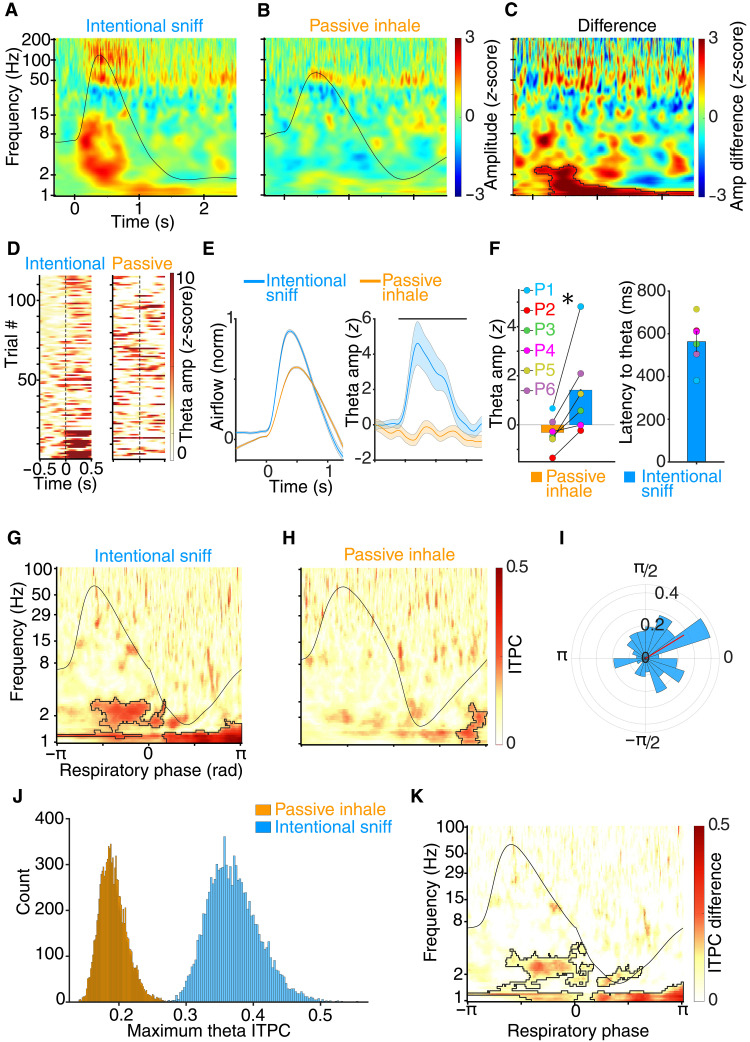
Intentional sniffs enhance and align theta oscillations in the olfactory bulb. (**A**) Sniff-onset-aligned amplitude spectrogram, averaged across participants during intentional sniffs. Black overlay: average respiratory signal. (**B**) Nasal-inhale-onset-aligned amplitude spectrogram, averaged across participants during passive breathing. (**C**) Difference between (A) and (B) (*N* = 6 participants, black outline indicates significance, permutation test, cluster corrected, *P* < 0.05). (**D**) Single-trial raster plots of theta amplitude for intentional sniffs (left) and passive inhales (right). Inhale onset at time 0. (**E**) Nasal airflow (left) and theta amplitude (right), averaged across participants during intentional sniffs (blue) and passive nasal inhales (orange) (*N* = 6 participants, black bar indicates significance, *P* < 0.05, cluster-based permutation test). (**F**) Left: Average theta amplitudes during intentional sniffs (blue) and passive inhales (orange), for the 500-ms time period following sniff onset (*N* = 6 participants, P1 to P6, *:P < 0.05, *T*_5_ = 3.17, *P* = 0.0247). Right: Latency to peak theta across participants (P1 to P6). (**G**) ITPC spectrogram for intentional sniffs, showing significant increases at theta frequency (*N* = 115 trials, black outline indicates significance, *P* < 0.05, cluster-corrected permutation test). (**H**) ITPC spectrogram for passive inhales (*N* = 115 trials, black outline indicates significance, *P* < 0.05, cluster-corrected permutation test). (**I**) Rose plot showing the distribution of single-trial theta phase values at the point of maximal ITPC from (G). Red line: mean vector. (**J**) Resampled distributions of ITPC values calculated using a random subset of trials (*N* = 95), for intentional sniffs (blue) and passive inhales (orange). Distributions were significantly different (mean ITPC difference ± 95% bootstrap CI = 0.1794 ± [0.1785, 0.1803]). (**K**) Difference in ITPC between intentional sniffs and passive nasal inhales (*N* = 6 participants, black outline indicates significance, *P* < 0.05, cluster-corrected permutation test of Fisher *Z*-transformed ITPC difference values).

The rodent sniff cycle is tracked by olfactory bulb respiratory oscillations overlapping with theta frequency (*29*). Each sniff, and therefore each respiratory/theta cycle, coordinates firing of olfactory bulb principal neurons (mitral/tufted cells). If the human olfactory bulb engages a similar mechanism for odor processing via theta oscillations, then we would expect a single human sniff to both increase the magnitude of theta oscillations and to reset their phase to align with the timing of sniff onset. To test this, we compared time-frequency analyses of olfactory bulb LFPs during intentional sniffs to those during passive nasal inhalations (Fig. 4, A to C). We found that intentional sniffs increased the amplitude of olfactory bulb theta oscillations (Fig. 4A), whereas passive inhales did not (Fig. 4B). A direct comparison revealed a significantly greater theta amplitude following inhale onset for intentional sniffs (Fig. 4C) (permutation test against amplitude differences, cluster-corrected *P* < 0.05). This effect was evident at the single-trial level (Fig. 4D). We further calculated the trial-averaged amplitude of sniff-aligned, theta-filtered (2 to 8 Hz) time series and found that theta amplitudes were significantly greater during intentional sniffs compared to passive inhales (Fig. 4E) (*P* < 0.05, cluster-based permutation test). This effect was consistent across individuals and was observed in every participant (Fig. 4F, left) (*T*_5_ = 3.17, *P* = 0.0247). The latency to maximum theta amplitude ranged from ∼380 to 720 ms (Fig. 4F, right).

If human olfactory bulb theta is analogous to rodent bulb theta, which is aligned and timed according to each sniff, then single human sniffs should coordinate the timing of theta oscillations in the bulb. One mechanism by which this could be accomplished is through resetting of the phase of theta oscillations with each sniff. To determine whether this occurs, we used intertrial phase coherence (ITPC)—which measures the consistency of instantaneous phase values over event-locked trials (*58*–*60*)—using inhale onset as the alignment point, to measure the consistency of the instantaneous phase of theta across trials of intentional sniffs versus trials of passive nasal inhalations. We found that, at the onset of an intentional sniff, the phase of olfactory bulb theta oscillations shifts such that alignment is consistent across single trials (Fig. 4G) (permutation test, cluster-corrected *P* < 0.05). This did not occur during passive inhalations (Fig. 4H) (permutation test, cluster-corrected *P* > 0.05). Phase shifting of theta was evident at the single-trial level (Fig. 4I) and significantly greater during intentional sniffs compared to passive inhales (Fig. 4, J and K). Specifically, using a resampling approach, we found that the maximum theta ITPC was significantly larger for intentional sniffs than for passive inhalations (Fig. 4J) {mean ITPC difference ± 95% bootstrap confidence interval (CI) = 0.1794 ± [0.1785, 0.1803]}. A direct statistical comparison revealed a significant difference in ITPC during intentional sniffs compared with passive inhalations (Fig. 4K) (*P* < 0.05, cluster-corrected permutation test of Fisher *Z*-transformed ITPC difference values).

### Sniffing behavior covaries with olfactory bulb theta oscillations

**Fig. 5.**
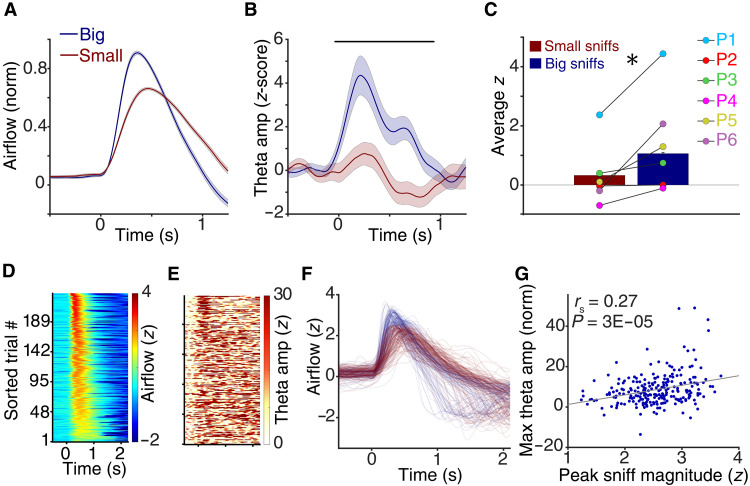
Sniffing behavior covaries with olfactory bulb theta oscillations. (**A**) Average time series of respiratory airflow for sniffs that have been ranked by maximal airflow and split into two groups. Solid lines are the mean, and shaded areas are ± SEM. (**B**) Average theta amplitude following sniff onset (time = 0) for big sniffs (dark blue) and small sniffs (dark red), showing a significant increase in theta amplitude for larger sniffs (*N* = 362 trials, two-tailed paired *t* test over time, cluster-corrected *P* < 0.05, trial-level cluster-based permutation test). Solid lines are the mean, and shaded areas are ± SEM. (**C**) Bar plots showing average theta amplitudes across participants (colored dots are individual participants) corresponding to the window from time = 0 to 1 s post-sniff, showing that theta is increased in response to stronger sniffs (*N* = 6 participants, two-tailed paired *t* test, *T*_5_ = −2.87, *P* = 0.035). Error bars are ± SEM. The asterisk (*) denotes statistical significance. (**D**) Raster plot of single-trial airflow values sorted by maximal airflow, plotted as color scale. (**E**) Raster plot of single-trial theta amplitudes corresponding to the trial order shown in (D), with evident decreases in theta amplitudes corresponding with decreases in sniff sizes. (**F**) Single-trial sniff traces of all trials shown in (A), (D), and (E), sorted by maximal airflow. Blue lines correspond to the larger half, and red lines correspond to the smaller half. (**G**) Scatterplot of single-trial values showing the correlation between the peak sniff magnitude and maximal theta amplitude (*N* = 362 trials, *r*_s_ = 0.27, *P* = 0.00003).

We next explored whether features of theta oscillations were related to features of intentional sniffs. For example, we might expect that, similar to rodents, a stronger or faster sniff would elicit stronger or faster theta oscillations in the bulb. To explore this, we first sorted trials by sniff size, as measured by maximal airflow (Fig. 5A), and then calculated theta responses for big and small sniffs separately. We found that, on average, sniffs with higher airflow corresponded to greater theta amplitudes (Fig. 5B) (two-tailed paired *t* test over time, cluster-corrected *P* < 0.05, trial-level cluster-based permutation test), an effect that was evident at the individual level (Fig. 5C) (*T*_5_ = −2.87, *P* = 0.035). These effects were also apparent in sorted single trials, where decreases in theta amplitude were observable when sorted according to decreasing sniff size (Fig. 5, D and E). In line with these effects, we found a positive correlation between the magnitude of sniffs of clean air and the maximal theta amplitude across trials (Fig. 5, F and G) (*r*_s_ = 0.27, *P* = 0.00003).

Because we found that intentional sniffs, but not passive nasal inhalations, increased theta power and, at the same time, that sniff magnitude was positively correlated with theta amplitude, it is possible that sniff kinematics contributed to the observed differences between intentional sniffs and passive inhales. To control for this possibility, we computed summary statistics of sniff peak flow, inhalation duration, and time-to-peak during both intentional sniffs and passive inhalations across participants and used a linear mixed-effects model to show that all measures significantly differed across conditions (fig. S4A). However, sniff duration contained some trials with overlapping values for both intentional sniffs and passive inhales (fig. S4B). This provided an opportunity to recompute spectrograms and ITPC for intentional and passive conditions using only the subset of trials for which sniff duration was matched. We found that, with sniff duration held constant, intentional sniffs still elicited stronger (fig. S4C), phase-aligned theta oscillations, whereas passive inhalations did not (fig. S4, D and E). We further found that, within the passive inhalation condition, there was no relationship between inhale size and theta magnitude (fig. S4F), in contrast to the relationship we observed during intentional sniffing. Together, these results indicate that intention to sniff drove the increased magnitude and phase alignment of theta oscillations.

### Theta oscillations organize the timing of gamma bursts in the olfactory bulb

**Fig. 6.**
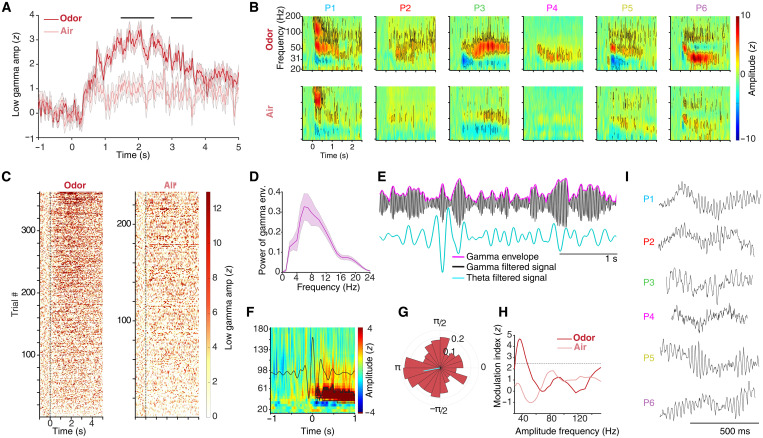
Theta oscillations organize the timing of gamma bursts in the olfactory bulb. (**A**) Average low gamma amplitude time series aligned to sniff onset (time = 0) for sniffs of odor and air (*N* = 6 participants, cluster-corrected *P* < 0.05, participant-level cluster-based permutation test). Black bars: statistical significance. (**B**) Amplitude spectrograms (ranging from 20 to 200 Hz) for each participant in response to odor (top row) and air (bottom row). Black outline: statistical significance (*N* = 6 participants, permutation test against pre-sniff baseline per participant, FDR-corrected at *P* < 0.05). (**C**) Raster plots of single-trial low gamma amplitude aligned to sniff onsets (time = 0) during sniffs of odor (left) and air (right). (**D**) Average power spectra of the low gamma envelope, showing fluctuations at theta frequency (*N* = 362 trials, *T*_361_ = 10.76, *P* = 1.3 × 10^−23^, two-sided paired *t* test of maximal power against zero). Shaded area: ± SEM. (**E**) Example of low-gamma-filtered signals (black) with the amplitude envelope (magenta) and theta-filtered signal (cyan) showing alignment between theta phase and gamma amplitude. (**F**) Theta-peak-aligned spectrogram averaged across participants showing gamma increases aligning to theta troughs. The black overlaid line is average theta-filtered LFP time series. Black outline: significance (*N* = 6 participants, permutation test against pre-sniff baseline, FDR corrected at *P* < 0.05). (**G**) Rose plot of low gamma bursts relative to theta phase at the single-trial level. Low gamma bursts show a phase preference for the trough of theta oscillation (*N* = 362 trials, *P* = 0.014, Rayleigh’s test). Cyan line: mean vector. (**H**) Modulation index for the theta phase of gamma amplitude, for sniffs of odor and air (*N* = 362 trials, permutation test against surrogate data, FDR corrected at *P* < 0.05, dashed line indicates FDR-corrected significance threshold). (**I**) Examples of gamma bursts occurring at theta troughs, evident in raw data from six participants.

Olfactory bulb gamma oscillations are associated with the presence of odor (*38*–*44*), have been linked to olfactory behaviors across species (*26*), and contribute to odor processing and concentration-invariant identity coding (*34*, *38*, *61*). Thus, gamma oscillations are a signature of odor processing in the olfactory bulb. If theta rhythms organize the timing of odor processing in the olfactory bulb, we would expect theta oscillations to coordinate the timing of gamma oscillations (30 to 150 Hz). In line with prior work (*40*–*46*), we found that increased olfactory bulb low gamma oscillations (30 to 60 Hz) were consistently associated with the presence of odor (Fig. 3, A to E). We further explored the consistency of odor-induced gamma oscillations and found that this narrowband, low gamma oscillation increased in magnitude when odor was present (Fig. 6A) (paired *t* test over time, cluster-corrected *P* < 0.05, participant-level cluster-based permutation test), in every participant (Fig. 6B) (permutation test against pre-sniff baseline, FDR corrected at *P* < 0.05), and that this increase was evident in single trials (Fig. 6C). Having established that gamma oscillations consistently emerge with odor, we next explored the relationship between theta phase and gamma amplitude, through a series of complementary analyses. First, we tested whether odor-induced gamma oscillations exhibited amplitude modulations in the theta range, by computing the average power spectrum of the gamma envelope across sniff-aligned odor trials, calculated for each trial using the irregular resampling autospectral analysis (IRASA) (*62*) to isolate oscillatory components of power. We found that the gamma envelope exhibited amplitude modulation in the theta range (Fig. 6, D and E) (*T*_361_ = 10.76, *P* = 1.3 × 10^−23^, two-sided paired *t* test of maximal power values against zero), peaking at 5.86 Hz. Second, we conducted a gamma-focused time-frequency analysis using sniff-induced theta peaks as the alignment point. If theta phase modulates gamma amplitudes, then we would expect a theta-locked increase in gamma power to emerge. We found that a significant increase in the amplitude of low gamma emerged at the trough of theta oscillations (Fig. 6F) (permutation test against pre-theta baseline, FDR-corrected significance threshold at *P* < 0.05 = 3.05). Third, we examined the relationship between gamma bursts and theta oscillations at the single-trial level. We computed the instantaneous phase of theta corresponding to each gamma burst and found a skewed distribution of phase values such that gamma peaks tended to occur on theta troughs (Fig. 6G) (*P* = 0.014, Rayleigh’s test). Fourth, we used a standard measure of phase-amplitude coupling, modulation index (*63*–*65*), to confirm these effects (Fig. 6H) and further confirmed that this measure decreased in magnitude in response to air (Fig. 6H) and on contacts distal versus proximal to the olfactory bulb (fig. S5). These effects were evident in the raw data at the single-trial level (Fig. 6I). Together, these results suggest that sniff-onset-aligned theta oscillations in the olfactory bulb act as a temporal window that organizes timing of odor processing, allowing for embedding of fine temporal resolution in a single sniff.

## DISCUSSION

On the basis of decades of robust research, the sniff has emerged as a key organizational unit of olfactory processing (*4*, *7*, *9*, *22*, *23*, *66*, *67*). In most mammals, precise sequences of sniff-phase-tuned activity across the olfactory bulb are evoked by rapid sniffing bursts during odor sampling (*68*). However, in an apparent exception to this rule, humans typically sample odors with slow, single sniffs. Whether this difference in sampling behavior implies differences in the basic mechanisms of human odor processing, including differences in the timing of neural oscillations across the olfactory bulb, has been unknown. Here, we recorded neural activity at high precision from the olfactory bulb in healthy volunteers and showed that intentional sniffs enhance and align theta oscillations, which, in turn, organize the synchronized firing of olfactory bulb neurons, measured through gamma oscillations at the single-trial level. Because rodent sniffs occur at rates overlapping theta frequency and align a ubiquitous theta oscillation in the rodent bulb (*29*), our findings suggest that human sniffs induce similarly timed neural oscillations in the human bulb. Thus, the theta oscillation may serve as a conserved rhythm of olfactory processing in mammalian olfaction, despite differences in sampling rates across species.

Theta oscillations have long been considered as a potential clocking mechanism across the mammalian brain (*69*–*72*). They are thought to play a functional role in both the organization of neural responses and in long-range communication through phase-referenced modulation of higher-frequency oscillations and coherence (*65*, *73*–*76*). Oscillatory synchrony and relationships between low-frequency reference frames and higher-frequency information processing appear to be a fundamental mechanism of neural circuit function that is conserved across vertebrates and nonvertebrates (*26*, *77*–*83*). These ideas may apply to neural oscillations in the olfactory system (*34*, *84*–*87*), but the tight link between theta oscillations in the olfactory bulb and sniffing behavior in rodents has limited our ability to disentangle contributions from each rhythm. Although the relationship between sniffing and odor processing is well established, the functional contribution of olfactory bulb theta oscillations is less understood. Given that sniffing and theta in the bulb are not always aligned (*7*, *88*–*92*), it is important to delineate the contributions of both rhythms to odor processing. Taking advantage of slower sniffing behavior in humans, we were able to isolate theta oscillations from sniff cycles and identified a relationship between theta phase and gamma amplitude in the olfactory bulb. We found that although gamma oscillations in the human olfactory bulb are still sniff linked, increasing at particular phases of the sniff/breath, there is also a faster modulatory rhythm, in the theta range, upon which gamma amplitudes ride. Thus, odor processing in the olfactory bulb can be simultaneously modulated by both respiratory rhythms and theta oscillations.

Studies suggest that the rodent olfactory bulb exhibits two distinct theta rhythms—one that is tightly locked to respiration and one, which is not always respiration locked, that originates from top-down sources like the hippocampus (*89*, *93*). During olfactory-driven tasks, olfactory bulb theta band activity or the sniff rhythm itself can be strongly coherent with hippocampal theta, depending on the task (*94*, *95*). In addition, during foraging and other spontaneous behaviors, hippocampal origins of olfactory bulb theta rhythms are evident (*89*, *93*, *96*). However, the origins of human olfactory bulb theta oscillations are unknown, and our data do not speak to their source. We can speculate here on potential sources.

One possibility is that human olfactory bulb theta originates from top-down sources such as the piriform cortex or hippocampus and is modulated by attentional factors during intentional sniffs. In line with this possibility, the piriform cortex exhibits theta oscillations during natural breathing (*97*) and odor sampling (*98*–*101*), and the phase of low-frequency oscillations in the piriform cortex is reset before attended sniffs (*102*). These findings dovetail with our result that intentional (and therefore attended) sniffs shift the phase of theta oscillations in the olfactory bulb post-sniff rather than pre-sniff and that odor cues elicit theta oscillations in the olfactory bulb.

Another speculative possibility is that human olfactory bulb theta oscillations originate from the periphery. Like other sensory systems, the olfactory system wants rhythmicity of stimuli (*103*): Across species and phyla, odors are drawn to olfactory receptors with rhythmicity, which is thought to contribute to odor processing (*104*). Rodents and mammals sniff in rhythmic bouts to draw odors through the nose, insects beat their wings to impose rhythm on odor sampling (*105*, *106*), lobsters wave their antennae (*107*), and fish cough and rhythmically pull aqueous odors to receptors (*108*). What adaptive feature might humans have to provide rhythmicity to odor sampling during a single, long sniff? We can think of two possibilities, neither of which has been explored. One is beating cilia within the nasal epithelium, which have been reported in the respiratory epithelium of humans (*109*), could impose a rhythmic pressure wave on an incoming air stream. The other is anatomical features of the upper nasal cavity, such as resonance properties or physical protrusions, spaced at distances such that air flowing past would be dynamically modulated at a theta frequency. For example, during a single fast nasal inhalation (or sniff), theta-spaced bumps near the olfactory cleft could impose fast paced “beats” of air onto mechanoreceptors, which are highly sensitive and could generate theta oscillations in response to even small modulations of air flow. A peripheral source of theta fits with our findings that features of sniffing, including sniff volume and duration, covary with features of olfactory bulb theta oscillations.

Both of these possibilities allow for a third scenario, in which there are two distinct human olfactory bulb theta oscillations with distinct origins, such as top-down and bottom-up sources, similar to rodents. Although beyond the scope of the current study, our data provide some qualitative hints that this may be the case. For example, we observed theta oscillations that range in frequency overlapping rodent theta and sniffing frequencies. It is possible that, as in rodents, olfactory bulb theta couples active sampling to ongoing theta oscillations in central brain regions during different behavioral states or task demands (*94*, *95*); additional studies are needed to determine whether this is the case.

A notable feature of our data is the presence of two distinct gamma oscillations in the olfactory bulb, each with different odor response properties and timing. Future work is needed to investigate these distinct responses, their relationships to respiration and the theta oscillation, and the extent to which they correspond with rodent bulb gamma responses. Gamma oscillations in the olfactory bulb of rodents have been studied extensively (*110*), perhaps reflecting differential activity in mitral/tufted cells (*5*, *41*, *111*), aspects of top-down and bottom-up influences on olfactory processing (*22*, *41*), the operations of multiple oscillatory networks (*40*, *61*, *112*), and emotional states (*113*). Because humans can report reliable olfactory perceptual information and experiences, the link we identify here between theta phase and gamma band amplitude suggests previously unexplored possibilities for understanding relationships between gamma oscillations and behavior in olfaction.

We note some limitations on interpretation of our work. It is possible that sniff kinematics, in addition to intention to sniff, contributed to the effects we observed. We were able to control for sniff duration across intentional and passive inhales (fig. S4), and we showed that there was no relationship between sniff speed and theta amplitude during passive inhales. These controls strongly support that the increase and alignment of theta we report were driven by intention to sniff rather than sniff features. We think it is likely that sniff kinematics contribute to shaping theta responses but only during intentional sniffing. Nonetheless, some sniff kinematics differed across conditions and cannot be completely ruled out as contributing factors. Furthermore, although the impact of the presence of the electrode in the nasal cavity on sniffing behavior is likely minimal (fig. S6), potential effects cannot be fully ruled out. To confirm localization of the recorded signal to olfactory bulb, we conducted a number of control analyses (Figs. 2 and 3), including recording data from a congenitally anosmic patient with no olfactory bulbs, conducting modality-specific control experiments, comparing the strength of odor responses with distance to the olfactory bulb in both the anterior and posterior directions, and examining intracranial recordings from nearby orbitofrontal areas during similar tasks. Despite these controls, volume-conducted signals from nearby structures could plausibly contribute to our recordings. Last, our sample size was limited to 6 due to the need for clinical time and space. To account for this, we conducted analyses at the single-participant and single-trial levels where possible. That said, our sample size limits our ability to generalize from our data characteristics of sniff-theta-gamma relationships across the broader population. Our data indicate that there may be significant interindividual variability in theta frequency responses, and there are established differences in sniffing behavior across individuals (*114*) and odor stimuli (*115*), which may lead to similar variability in theta-gamma coupling strength. Nonetheless, although these parameters may vary between individuals, our data suggest a consistent mechanism of low-frequency responses organizing higher-frequency responses in the olfactory bulb.

The field of olfaction has focused on the sniff rhythm, more so than the theta rhythm, as the organizing structure of neural responses to odor. In our study, the theta oscillation emerged as an additional organizational unit of olfaction, acting as a processing component between the sniff and higher-frequency responses in the olfactory bulb.

## MATERIALS AND METHODS

### Participants

This study was approved by the Northwestern Institutional Review Board under protocol number STU00218720. Participation was voluntary, and written informed consent was obtained from each participant. The study was conducted according to the principles of the Declaration of Helsinki and the Belmont Report.

### Electrode preparation

Electrodes (Ad-Tech MM16A-SP05X-000) included six platinum ring contacts on a 1.3-mm-diameter urethane catheter; contacts are 1.57 mm wide, spaced 5 mm apart center-to-center (Fig. 3F). Before each recording session, electrodes were modified to improve impedance and directionality with application of a nonconductive conformational coating such that only the upper half of each ring contact was exposed. A small nasal sponge (Merocel, Medtronic) was used to stabilize the distal end of the electrode at the opening of the olfactory cleft. Measurement marks were placed on the surface of the electrode catheter, registered to the position of each participant’s nare entry, allowing for additional precision in placement and providing a reference for visual confirmation of electrode stability during recording.

### Electrode placement

On the basis of CT scan imaging and measurements of individual nasal anatomy, electrodes were placed under endoscopic guidance along the superior surface of the nasal cavity, just below the cribriform plate, sitting inside the nasal cavity on the olfactory epithelium, ∼1 mm away from the olfactory bulb, by an experienced ENT (coauthor B.K.T.) with expertise in human olfactory cleft anatomy. Electrodes were placed without anesthetic, as the process involves only brief minimal discomfort which resolves immediately when the electrode is in place. Decongestant (1% solution phenylephrine) is used to open up the anterior nasal cavity before electrode placement but is not applied to the olfactory cleft. Proper electrode positioning was confirmed by CT scan (Fig. 1C). Surface electrodes were placed at the right and left mastoids for reference signals and at the scalp surface location Fz for ground.

### CT and MRI

CT scans were acquired with a cone beam CT machine (Xoran) with an ultralow-dose CT scan (CTDIvol = 1.3 milligray; patient effective dose estimate = 0.04 mSv) sinus-scan protocol. The voxel size was 0.4 mm. MRIs were acquired with a Siemens 3T Prisma scanner with a 64-channel head-neck coil. T1-weighted structural scans were acquired with the MPRAGE protocol at a 1-mm isotropic resolution [echo time (TE) = 2.98 ms, repetition time (TR) = 2.3 s, flip angle = 9°, and image size = 208 by 240 by 256 voxels]. T2-weighted structural scans were focused on the area around the olfactory bulbs and were acquired with the Siemens T2 SPC ZOOMit protocol at a 0.5-mm isotropic resolution (TE = 125 ms, TR = 1 s, flip angle = 100°, and image size = 320 by 164 by 164 voxels).

### Neural and respiratory recordings

Intranasal electrode tails were connected to a lightweight electrode connection system (Ad-Tech CABRIO L-SRL-10DIN). Surface and intranasal electrode signals were acquired with a Neuralynx ATLAS neurophysiology system (ATLAS Acquisition Amplifier 31-0605-0139). Respiratory signals were recorded with a pneumotachometer (Hans Rudolph) connected to a mask placed over the nose and mouth (Philips Respironics Amara Silicone Cushion, 1090294) and recorded directly into the ATLAS system via dc input. All recordings were sampled at 2000 Hz. Data were acquired using a mastoid electrode (lowest impedance) as a reference and Fz as ground.

### Behavioral tasks

All participants were confirmed to be normosmic via the UPSIT (*116*), with the exception of the congenital anosmic participant who was confirmed anosmic via the UPSIT. Behavioral tasks included odor detection, cue/stimulus matching of odors, cue/stimulus matching of images, cued sniffing of medical-grade air, and passive nasal inhalation of medical-grade air. Participants were fitted with a mask for odor delivery and respiratory monitoring. Experimental tasks were computer controlled, presented on a monitor positioned in front of the participant mirroring a laptop computer (Apple) running MATLAB (MathWorks Inc., RRID:SCR_001622) via the PsychToolBox extension (RRID:SCR_002881). Synchronization transistor-transistor logic (TTL) pulses were delivered to the ATLAS system using a data acquisition board (USB-1208FS, Measurement Computing) to mark task events, including participant responses. Odors were delivered using a 12-channel computer-controlled, air-dilution olfactometer through polytetrafluoroethylene (PTFE)–lined tubing connected to the bottom of the mask, in a continuous stream of clean air flowing at 6 standard liters per minute (SLPM). Odors were cleared from the mask between odor presentations using a vacuum line connected to the top of the mask.

#### Odor detection task

On each trial, participants sniffed either phenylethyl alcohol (PEA) or clean air, in randomized order. On 50% of the trials, the stimulus was PEA, and on the other 50%, the stimulus was clean air. Each trial began with a 3-s audiovisual countdown to a prompt to sniff. The sniff prompt lasted for 2 s, after which the question “Was there an odor present?” appeared, with “Yes” or “No” as possible answers, indicated by button press with random button orientations. This task included 40 trials.

#### Cue-matching tasks

The cue-matching tasks were designed to test whether participants were able to match a visual and spoken word cue with a later stimulus. Each trial began with audiovisual presentation of a word cue that corresponded to 1 of 10 possible stimuli, which were presented following the cue after a jittered delay, in randomized order. Participants were then asked whether the stimulus matched the cue and responded by button press. One version of this task used olfactory stimuli (PEA, sweet orange extract, coffee beans, methyl salicylate, peanut butter, garlic extract, chocolate extract, coconut oil, parmesan cheese, and amyl acetate), and the other used visual stimuli (images of a cat, chair, coffee mug, dog, elephant, lady bug, lamp, laptop, spoon, and toothbrush). Task versions were otherwise identical.

#### Intentional sniff task

Each trial began with a 3-s audiovisual countdown to sniff. Participants were instructed to take a single sniff at the cue with no additional instructions on how to sniff. Participants performed 20 trials with a 10-s inter-stimuli interval (ISI).

#### Passive nasal inhalation task

To measure the effects of passive breathing, participants were instructed to relax and breathe through the nose for 5 min while we acquired baseline data.

### Preprocessing

For each participant, electrophysiological data were downsampled to 500 Hz, and electrode signals were notch filtered at 60 Hz and its harmonics up to 180 Hz. The respiratory signal was mean subtracted and low-pass filtered at 10 Hz. Intranasal electrode contacts were locally re-referenced to the neighboring electrode contact (i.e., bipolar reference, except for P2 and P4 where the mastoid reference was used due to technical issues). A single electrode contact that was in closest proximity to the olfactory bulb of each participant was used for all analyses. All sniff and stimulus onsets were marked using a custom in-house graphical user interface.

### Data analyses

Data analyses were conducted in MATLAB (MathWorks Inc., RRID:SCR_001622) using custom code and functions from the FieldTrip toolbox and the CircStat toolbox (*117*). All analyses were performed at both the group and the individual participant level.

### Amplitude spectrograms

Spectrograms were computed using MATLAB (MathWorks Inc., RRID:SCR_001622) with custom code and functions from the FieldTrip toolbox (*118*) (RRID:SCR_004849). A two-pass, zero-phase-lag, finite impulse response (FIR) filter was used for filtering, as implemented in FieldTrip. Amplitude spectrograms were computed using a filter-Hilbert approach (*60*, *119*). We filtered preprocessed LFP data from 1 to 200 Hz in 100 logarithmically spaced frequencies with bandwidth increasing from 2 to 30 Hz in 100 logarithmically spaced frequencies. The FIR filter order was determined automatically inside the ft_preproc_bandpassfilter function of the FieldTrip toolbox. On the basis of our filtering parameters, we used FIR filter orders that ranged from ∼55 to 825 taps. At a sampling rate of 500 Hz, this means the shortest filter length was 0.11 s, and the longest filter length was 1.65 s. The Hilbert transform of the filtered signals was used to calculate the amplitude envelope, which was temporally smoothed with a moving average filter kernel of 10 ms, segmented into epochs 1 s before and 5 s after inhale onset time points, and averaged at each frequency. Amplitude values were normalized to a baseline period of 100 to 600 ms before inhale onset. A permutation method (*119*) was used to determine statistical significance. Specifically, time points of inhale onsets were circularly shifted in time by a random amount while maintaining the relative distance between them, amplitude was calculated, and this was repeated 10,000 times, resulting in a null distribution of baseline amplitude. The SD of the baseline amplitude was used to divide the baseline-normalized real spectrogram to create a *z*-score map. Individual participant spectrograms were used in Figs. 2B and 5B. In all other cases, spectrograms were combined across participants in two ways: calculating amplitude spectrograms for each individual participant then calculating statistics on the participant-wise-average spectrogram or calculating a spectrogram on data concatenated across participants. Participant-wise-average spectrograms were used in the following figure panels: Figs. 3 (B and I) and 4 (A to C) and fig. S4C. Spectrograms calculated on data concatenated across participants were used in the following figure panels: Figs. 2 (C and F), 3 (A and C), 4 (A to C), and 6F and figs. S2 and S3.

### Intertrial phase coherence

ITPC (*59*, *60*) was used to measure phase reset of oscillations (Fig. 3, G to K). First, respiratory epochs from −0.6 to 2 s around inhale onset were generated for two conditions: intentional sniffs and passive inhalations. To address differences in number of trials by condition, a random subset of passive inhales, matched in trial number to intentional sniffs, was analyzed within each participant. To control for temporal differences in breathing during these two conditions, respiratory epochs were normalized by phase, such that a single breath corresponding to each trial was separated into 200 equally spaced respiratory phase bins. Next, single-trial epochs of LFPs centered around inhale onset with a time window of −0.6 to 2 s were generated for the two conditions and were then binned into the 200 respiratory phase bins. ITPC was then calculated across trials by condition on the respiratory-phase-normalized epochs of LFPs by calculating the consistency in phase across trials over time, as measured by the length of the mean vector, using the following equation

ITPC=abs(mean(exp(1i∗k)))

where **k** is a vector of phase angles at one time-frequency point over trials (*59*, *60*). The significance of ITPC within condition (Fig. 4, G to K) was assessed using a permutation approach. Specifically, the phase epochs of each condition were circularly shifted in time by a random amount, and ITPC was calculated. This was repeated 1000 times to create a null distribution of ITPC values per condition. Cluster-based correction was used to establish time-frequency clusters that were significantly above the permuted distribution. The phase of each trial at maximal ITPC at theta frequency was extracted for the polar histogram plot in Fig. 4I. The difference in ITPC between conditions was assessed with a combined approach. A bootstrap resampling approach (Fig. 4J) yielded resampled distributions of ITPC across the two conditions, by resampling the order of the epochs with replacement over 10,000 iterations, with the 95% CI calculated on the difference between the two resampled distributions. In an additional approach, the difference in ITPC values between conditions was calculated and Fisher *Z*-transformed (*60*), and significance was assessed using a permutation approach (Fig. 4K). The phase epochs within each condition were circularly shifted over time, the difference in ITPC was calculated (*60*), and this process was repeated 1000 times to create a null distribution of ITPC difference values.

### Cross-frequency coupling of theta and gamma

To assess cross-frequency coupling between theta and gamma, we used a combination of analyses. The power of the gamma envelope (Fig. 6D) was calculated by filtering LFPs at low gamma frequency (30 to 60 Hz), extracting the amplitude envelope Hilbert transform of the filtered signal, and calculating power using the IRASA to isolate oscillatory components of power (*62*). IRASA was calculated using the FieldTrip toolbox (ft_freqanalysis.m). The frequency resolution was 0.98 Hz and ranged from 0 to 150 Hz. The mean and SE of power was calculated across trials. An additional approach aligned LFPs to theta troughs (Fig. 6F). LFPs were filtered at theta frequency (2 to 8 Hz) and separated into epochs centered around sniff onsets. These were then *z*-scored, and theta peak and trough time points were detected by extracting the first theta peak and trough to cross a threshold of 1.45 *z* and −1.45 *z*, respectively. The time points of theta peaks and troughs across trials were used to calculate amplitude spectrograms as described above. A third approach detected single gamma bursts per trial and related these to theta phase (Fig. 6G), by detecting maximal gamma amplitude within the theta oscillatory time points detected in the previous analysis. First, LFPs were filtered at 30 to 60 Hz. Then, the low-gamma-filtered signals were epoched into individual trials at the theta time points detected in the approach described above. Peaks in the low-gamma-filtered epochs were detected by finding local maxima (islocalmax MATLAB function) of the low-gamma-filtered signal. Nonuniformity of gamma peaks relative to theta phase were assessed using the CircStat.m (*117*) statistics toolbox. A fourth approach used modulation index (Fig. 6H and fig. S5) (*63*) using methods described previously (*100*), isolating maximal modulation index at 4 Hz for phase-modulating frequencies across amplitude frequencies spanning the gamma band (30 to 150 Hz).

### Statistical analysis

MATLAB was used for statistical analysis. For amplitude spectrograms, a permutation method (*119*) was used to determine statistical significance, and FDR correction was used to correct for comparisons across time-frequency points. For amplitude difference spectrograms, condition group labels were shuffled to create a permuted distribution of differences, and significance was assessed following cluster-based correction. Paired two-tailed *t* tests were used for participant-level comparisons. Two-sample two-sided *t* tests were used for trial-level comparisons. Time-series comparisons were assessed for statistical significance using a cluster-based permutation test across time (5000 permutations). For within-participant time-series comparisons, condition differences were sign flipped at the participant level to generate the null distribution. Clusters were formed using a two-sided threshold of *P* = 0.2 and evaluated against the maximum cluster-mass distribution (cluster-level α = 0.05). For trial-level time-series comparisons, a *t*-statistic was computed across trials for each time point, and trial-level signs were randomly flipped to generate a permutation distribution, with clusters formed using a two-sided threshold of *P* < 0.05, and evaluated against the maximum cluster-mass distribution (cluster-level α = 0.05). A linear mixed-effects model with condition as a fixed effect and participant as a random intercept was used to compare breathing kinematics between intentional sniffs and passive inhalations, with estimated differences assessed using Wald tests. The number of experimental samples (*N*) in each group is indicated in the legend. Results are presented as means ± SEM. Threshold for significance was *P* < 0.05. Circular statistics were conducted in CircStat (Rayleigh test: circ_rtest.m, mean vector length: circ_r.m, and circular mean: circ_mean.m).

## Supplementary Materials

### This PDF file includes:

Figs. S1 to S6 Table S1 References
