# Supplementary material for: Theta oscillations are an organizational unit of odor processing in the olfactory bulb
Source: Sci Adv. 2026 Jul 3;12(27):eaee1002. doi: 10.1126/sciadv.aee1002 (PMC13330860; doi:10.1126/sciadv.aee1002)
Supplement: Supplementary file 1 — Figs. S1 to S6 Table S1 References [file sciadv.aee1002_sm.pdf]

Science Advances
AAAS

Supplementary Materials for

Theta oscillations are an organizational unit of odor processing in the olfactory bulb

Andrew Sheriff *et al.*

Corresponding author: Andrew Sheriff, andrew.sheriff@northwestern.edu;
Christina Zelano, c-zelano@northwestern.edu

Sci. Adv. **12**, eaee1002 (2026)
DOI: 10.1126/sciadv.aee1002

This PDF file includes:

Figs. S1 to S6
Table S1
References

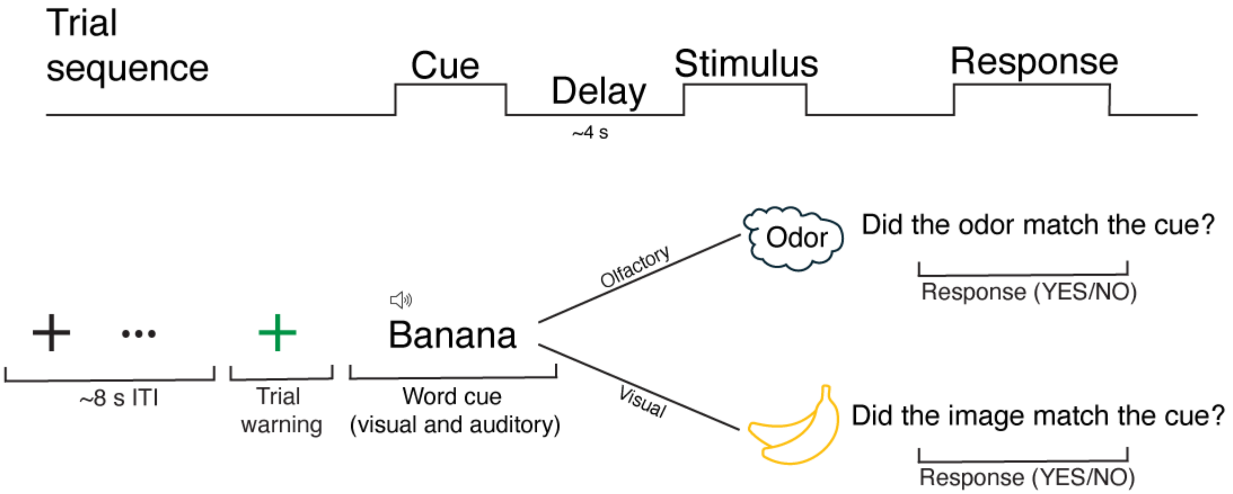

Fig. S1. Olfactory and visual cue-matching tasks experimental design

Experimental design was identical for olfactory and visual conditions, including cues, until presentation of stimulus. For both conditions, each trial began with an audiovisual cue consisting of a written and spoken word (e.g. banana), followed by a delay after which an odor was presented in the olfactory condition and a image was presented in the visual condition. Participants then indicated via button press whether the stimulus matched the cue. Therefore, an identical cue took on two different meanings: in one case indicating that an odor would arrive soon, and in the other case indicating that an image would arrive soon. Critically, the cues were otherwise identical, and no odor or image was presented during the cues.

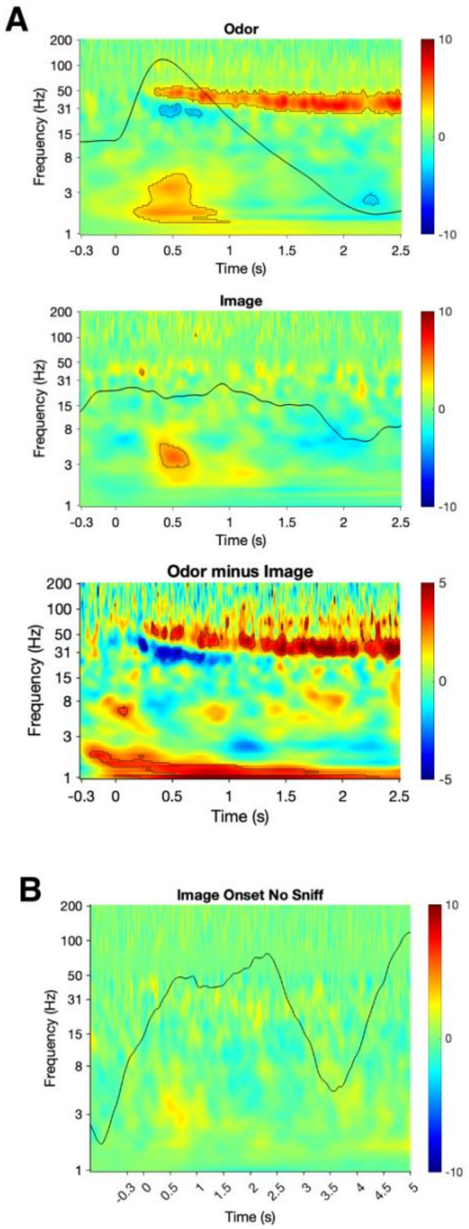

Fig. S2. Time-frequency amplitude spectrograms during olfactory vs. visual cue-matching tasks

(**A**) Time-frequency amplitude spectrograms for odor (top), image (middle), and their difference (bottom). A significant increase in theta and gamma amplitudes was found in response to odors compared to images. Statistical significance outlined in black ($P < 0.05$, $N = 240$ trials, permutation test of amplitude differences, cluster-corrected). (**B**) To determine whether the significant response to images in the theta range in A was driven by spontaneous alignment of sniffs with stimulus onset (*121*), we calculated the time-frequency amplitude spectrogram of responses to images with trials containing spontaneously aligned sniffs removed, showing no significant effects ($P > 0.05$, $N = 70$ trials).

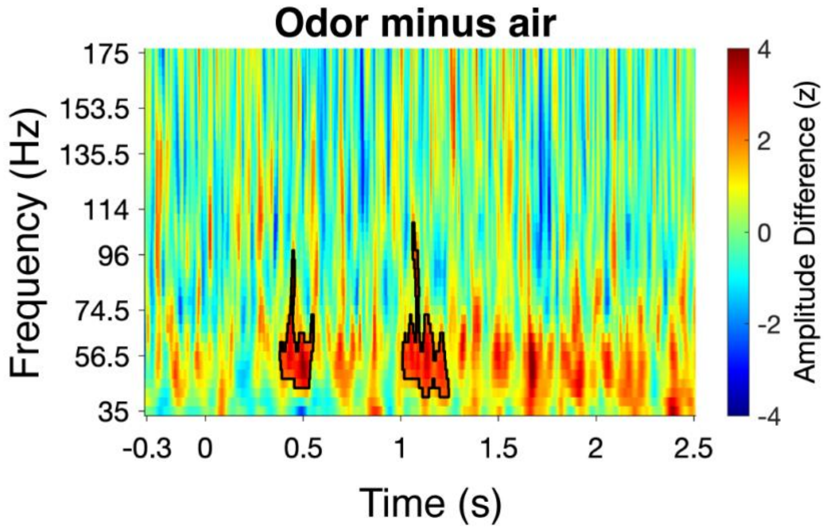

Fig. S3. Gamma amplitude was increased for odor compared to air on odor detection task
Difference in time-frequency amplitude spectrograms computed for odor minus air. Black outline indicates statistical significance ($P < 0.05$, $N = 122$ trials, permutation test, cluster corrected).

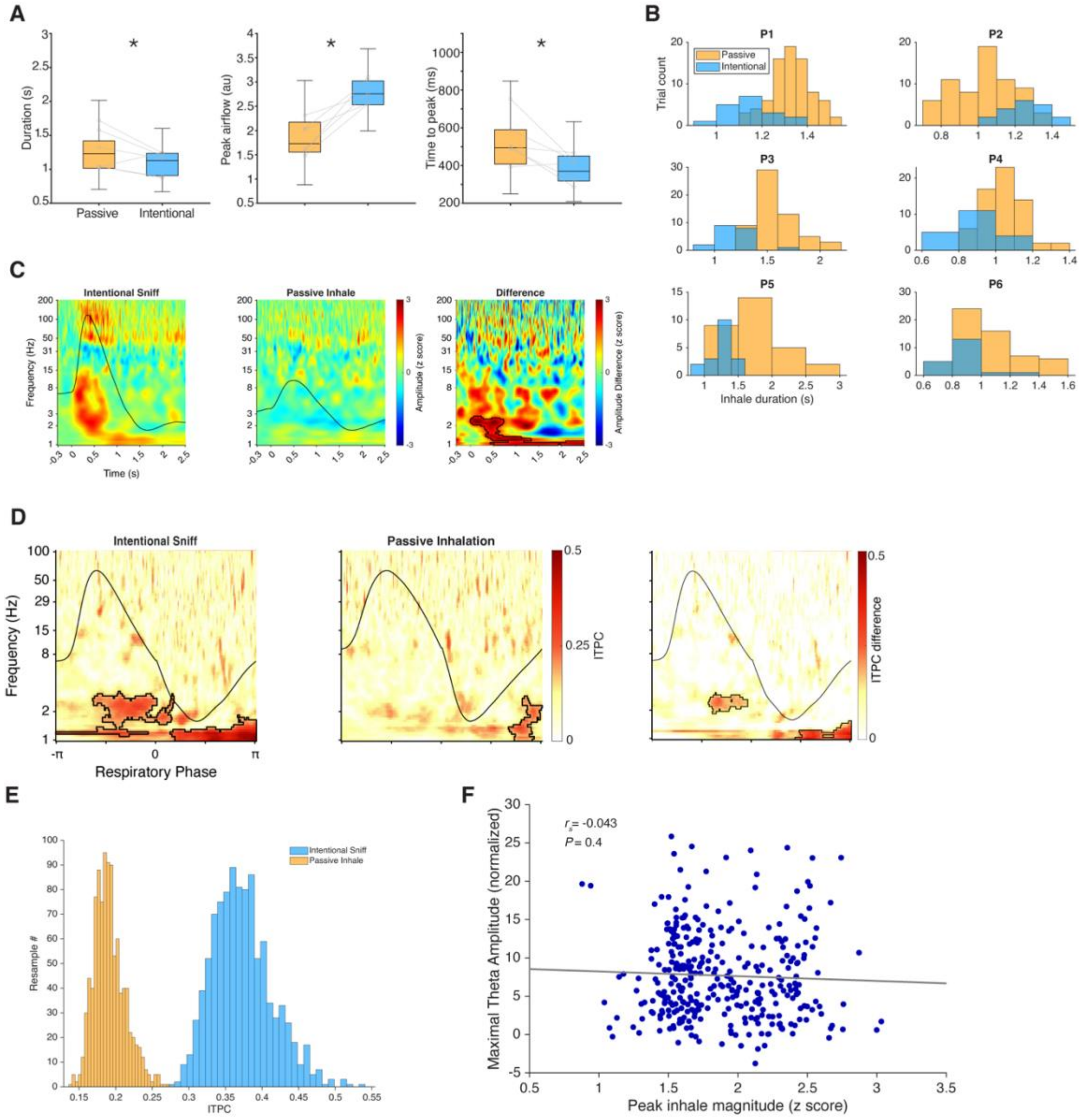

Fig. S4. Quantifying and controlling for kinematics of intentional sniffs compared to passive inhales

(**A**) Summary statistics of sniff kinematics showing inhale duration (left), airflow peak (middle), and time-to-peak (right) across participants. A linear mixed-effects model was used to compare kinematics between intentional sniffs and passive inhales, with estimated differences assessed using Wald tests (estimate ± SE). Inhale duration was significantly shorter for intentional sniffs compared to passive inhales, with an estimated difference of -0.176 ± 0.021 s (T_{471} = -8.19, P = 0.000, N = 472 trials). Peak airflow was significantly higher for intentional sniffs compared to passive inhales, with an estimated difference of 0.946 ± 0.035 au (T_{471} = 27.24, P = 0.000, N = 472 trials). Time to peak airflow was significantly lower for intentional sniffs compared to passive inhales, with an estimated difference of -136.93 ± 12.8 ms (T_{471} = -10.70, P = 0.000, N =

472 trials). (**B**) Binned histograms of inhale durations for intentional sniffs (blue) and passive inhales (orange). Histograms show trial counts of inhale durations falling within each bin, for each participant, with overlap between conditions shown in dark blue. (**C**) Time-frequency amplitude spectrograms computed on trials that were matched in inhale duration for intentional sniffs (left), passive inhales (middle) and their difference (right, $P < 0.05$, $N = 120$ trials, cluster-based permutation test). (**D**) Phase-by-frequency ITPC spectrograms for intentional sniffs (left), passive inhales (middle) and their difference (right), for trials that were matched in sniff duration. Black outline indicates significance ($N = 115$ trials, black outline indicates significance at $P < 0.05$ of cluster-corrected permutation test). (**E**) Resampled distributions of ITPC values computed on trials that were matched in inhale duration for intentional sniffs (blue) and passive inhales (orange). Distributions were significantly different (mean ITPC difference ± 95% bootstrap CI = 0.1798 ± [0.1771, 0.1825)]). (**F**) Scatterplot of single-trial theta amplitudes showing no significant correlation between passive inhale strength and theta amplitudes ($r_s = 0.043$, $P = 0.4$, $N = 358$ trials).

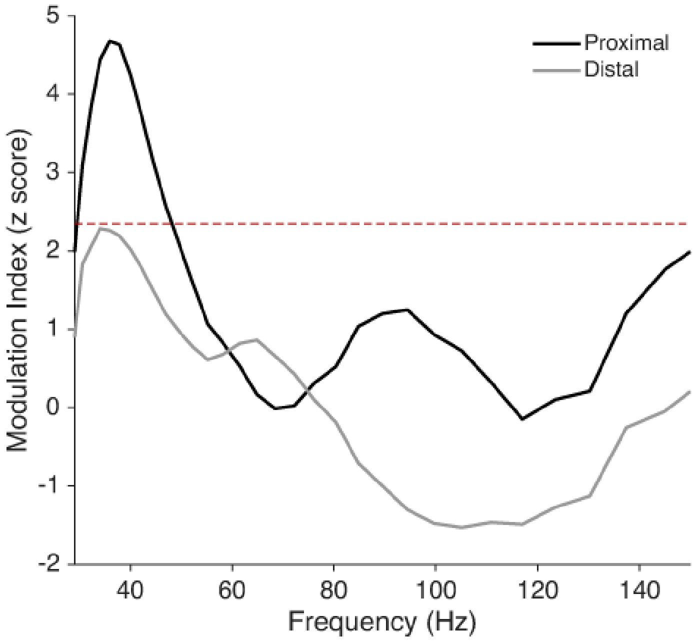

Fig. S5. Modulation Index for electrode contacts proximal and distal to olfactory bulb

Modulation Index of gamma amplitude by theta phase for sniffs of odor on proximal contacts (black line) and distal contacts (gray) (permutation test against surrogate data, FDR corrected at $P < 0.05$, red dashed outline indicates FDR corrected significance threshold, $N = 362$ trials).

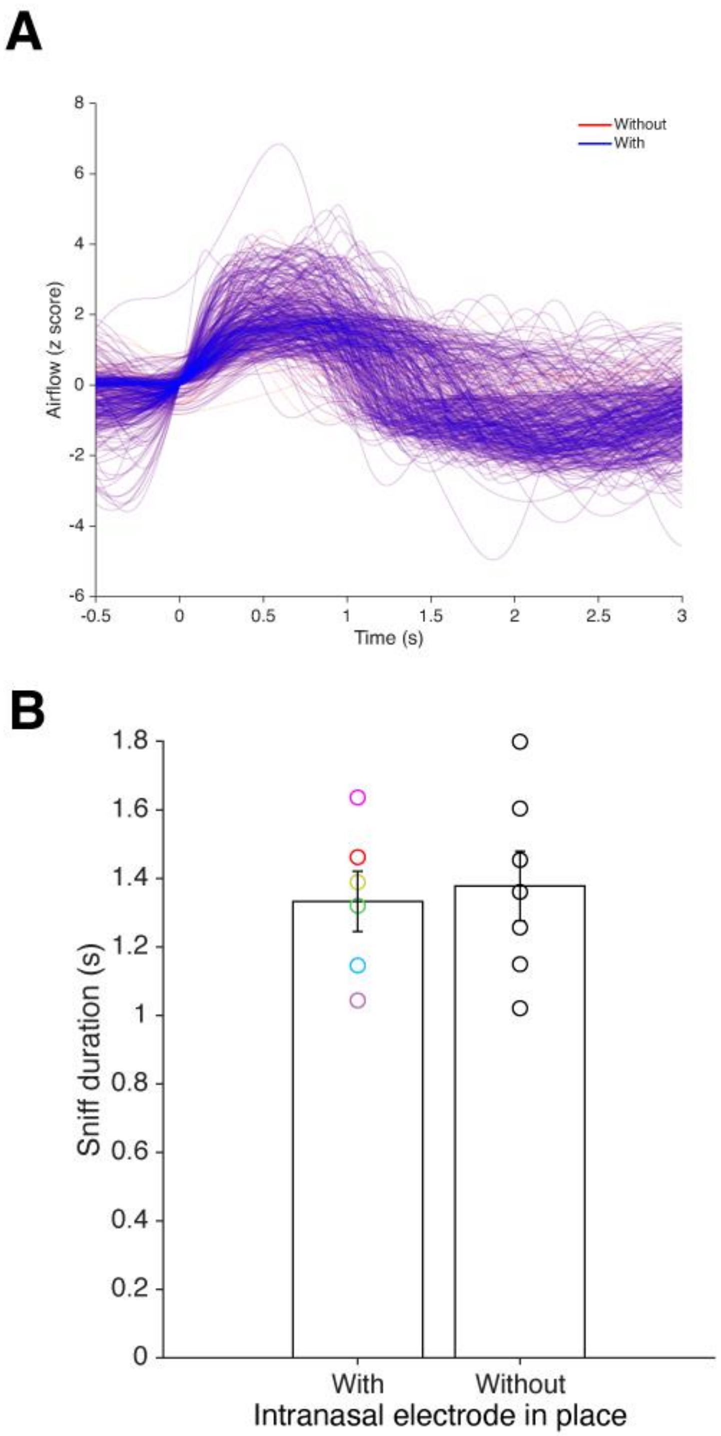

Fig. S6. Sniffs with and without intranasal electrode in place

(**A**) Nasal airflow during intranasal recordings with the electrode in place (blue) and nasal airflow during intracranial EEG recordings without the electrode in place (red), from a previous study (*55*). (**B**) Bar plot of durations of all sniffs taken with the electrode in place and all sniffs taken without the electrode in place. No significant difference was found (T_{11}, $P = 0.7474$, $N =$ 13 participants).

Table 1. Demographics table.

Participant	Age	Gender
P1	46	M
P2	26	M
P3	28	F
P4	24	F
P5	34	M
P6	24	M
Anosmic	31	M

REFERENCES

1. M. Wachowiak, All in a sniff: Olfaction as a model for active sensing. *Neuron* **71**, 962–973 (2011).

2. S. P. Mysore, E. I. Knudsen, Descending control of neural bias and selectivity in a spatial attention network: Rules and mechanisms. *Neuron* **84**, 214–226 (2014).

3. M. Szwed, K. Bagdasarian, E. Ahissar, Encoding of vibrissal active touch. *Neuron* **40**, 621–630 (2003).

4. R. Shusterman, M. C. Smear, A. A. Koulakov, D. Rinberg, Precise olfactory responses tile the sniff cycle. *Nat. Neurosci.* **14**, 1039–44 (2011).

5. H. Manabe, K. Mori, K. Mori, Sniff rhythm-paced fast and slow gamma-oscillations in the olfactory bulb: Relation to tufted and mitral cells and behavioral states. *J. Neurophysiol.* **110**, 1593–1599 (2013).

6. J. V. Verhagen, D. W. Wesson, T. I. Netoff, J. A. White, M. Wachowiak, Sniffing controls an adaptive filter of sensory input to the olfactory bulb. *Nat. Neurosci.* **10**, 631–639 (2007).

7. K. M. Cury, N. Uchida, Robust odor coding via inhalation-coupled transient activity in the mammalian olfactory bulb. *Neuron* **68**, 570–585 (2010).

8. R. Haddad, A. Lanjuin, L. Madisen, H. Zeng, V. N. Murthy, N. Uchida, Olfactory cortical neurons read out a relative time code in the olfactory bulb. *Nat. Neurosci.* **16**, 949–957 (2013).

9. M. Smear, R. Shusterman, R. O’Connor, T. Bozza, D. Rinberg, Perception of sniff phase in mouse olfaction. *Nature* **479**, 397–400 (2011).

10. M. Smear, A. Resulaj, J. Zhang, T. Bozza, D. Rinberg, Multiple perceptible signals from a single olfactory glomerulus. *Nat. Neurosci.* **16**, 1687–1691 (2013).

11. R. Iwata, H. Kiyonari, T. Imai, Mechanosensory-based phase coding of odor identity in the olfactory bulb. *Neuron* **96**, 1139–1152.e7 (2017).

12. X. Grosmaitre, L. C. Santarelli, J. Tan, M. Luo, M. Ma, Dual functions of mammalian olfactory sensory neurons as odor detectors and mechanical sensors. *Nat. Neurosci.* **10**, 348–354 (2007).

13. A. Fontanini, J. M. Bower, Slow-waves in the olfactory system: An olfactory perspective on cortical rhythms. *Trends Neurosci.* **29**, 429–437 (2006).

14. A. Fontanini, J. M. Bower, Variable coupling between olfactory system activity and respiration in ketamine/xylazine anesthetized rats. *J. Neurophysiol.* **93**, 3573–81 (2005).

15. M. E. Phillips, R. N. S. Sachdev, D. C. Willhite, G. M. Shepherd, Respiration drives network activity and modulates synaptic and circuit processing of lateral inhibition in the olfactory bulb. *J. Neurosci.* **32**, 85–98 (2012).

16. N. Onoda, K. Mori, Depth distribution of temporal firing patterns in olfactory bulb related to air-intake cycles. *J. Neurophysiol.* **44**, 29–39 (1980).

17. F. Macrides, S. L. Chorover, Olfactory bulb units: Activity correlated with inhalation cycles and odor quality. *Science* **175**, 84–87 (1972).

18. T. Ackels, R. Jordan, A. T. Schaefer, I. Fukunaga, Respiration-locking of olfactory receptor and projection neurons in the mouse olfactory bulb and its modulation by brain state. *Front. Cell. Neurosci.* **14**, 220 (2020).

19. M. A. Rosero, M. L. Aylwin, Sniffing shapes the dynamics of olfactory bulb gamma oscillations in awake behaving rats. *Eur. J. Neurosci.* **34**, 787–799 (2011).

20. L. M. Kay, "Circuit oscillations in odor perception and memory" in *Progress in Brain Research* (Elsevier B.V., ed. 1, 2014), vol. **208**, pp. 223–251.

21. N. Buonviso, C. Amat, P. Litaudon, Respiratory modulation of olfactory neurons in the rodent brain. *Chem. Senses* **31**, 145–154 (2006).

22. N. Buonviso, C. Amat, P. Litaudon, S. Roux, J. P. Royet, V. Farget, G. Sicard, Rhythm sequence through the olfactory bulb layers during the time window of a respiratory cycle. *Eur. J. Neurosci.* **17**, 1811–1819 (2003).

23. A. Kepecs, N. Uchida, Z. F. Mainen, The sniff as a unit of olfactory processing. *Chem. Senses* **31**, 167–179 (2006).

24. Y. Wu, K. Chen, C. Xing, M. Huang, K. Zhao, W. Zhou, Human olfactory perception embeds fine temporal resolution within a single sniff. *Nat. Hum. Behav.* **8**, 2168–2178 (2024).

25. G. Lane, G. Zhou, T. Noto, C. Zelano, Assessment of direct knowledge of the human olfactory system. *Exp. Neurol.* **329**, 113304 (2020).

26. L. M. Kay, Olfactory system oscillations across phyla. *Curr. Opin. Neurobiol.* **31**, 141–147 (2015).

27. H. L. Eisthen, Why are olfactory systems of different animals so similar? *Brain Behav. Evol.* **59**, 273–293 (2002).

28. L. M. Kay, L. R. Lancaster, W. J. Freeman, Reafference and attractors in the olfactory system during odor recognition. *Int. J. Neural Syst.* **7**, 489–95 (1996).

29. D. Rojas-Líbano, D. E. Frederick, J. I. Egaña, L. M. Kay, The olfactory bulb theta rhythm follows all frequencies of diaphragmatic respiration in the freely behaving rat. *Front. Behav. Neurosci.* **8**, 214 (2014).

30. L. M. Kay, J. Beshel, J. Brea, C. Martin, D. Rojas-Líbano, N. Kopell, Olfactory oscillations: The what, how and what for. *Trends Neurosci.* **32**, 207–214 (2009).

31. B. Gourévitch, L. M. Kay, C. Martin, Directional coupling from the olfactory bulb to the hippocampus during a go/no-go odor discrimination task. *J. Neurophysiol.* **103**, 2633–2641 (2010).

32. C. Martin, J. Beshel, L. M. Kay, An olfacto-hippocampal network is dynamically involved in odor-discrimination learning. *J. Neurophysiol.* **98**, 2196–2205 (2007).

33. F. David, E. Courtiol, N. Buonviso, N. Fourcaud-Trocmé, Competing mechanisms of gamma and beta oscillations in the olfactory bulb based on multimodal inhibition of mitral cells over a respiratory cycle. *eNeuro* **2**, ENEURO.0018–ENEU15.2015 (2015).

34. J. Losacco, D. Ramirez-Gordillo, J. Gilmer, D. Restrepo, Learning improves decoding of odor identity with phase-referenced oscillations in the olfactory bulb. *Elife* **9**, e52583 (2020).

35. C. Zelano, N. Sobel, Humans as an animal model for systems-level organization of olfaction. *Neuron* **48**, 431–454 (2005).

36. C. Sem-Jacobsen, R. Bickford, H. Dodge, M. Petersen, Human olfactory responses recorded by depth electrography. *Proc. Staff Meet. Mayo Clin.* **28**, 166–70 (1953).

37. J. R. Hughes, D. E. Hendrix, N. Wetzel, J. W. Johnston, Correlations between electrophysiological activity from the human olfactory bulb and the subjective response to odoriferous stimuli. *Electroencephalogr. Clin. Neurophysiol.* **28**, 97–8 (1970).

38. J. Beshel, N. Kopell, L. M. Kay, Olfactory bulb gamma oscillations are enhanced with task demands. *J. Neurosci.* **27**, 8358–8365 (2007).

39. L. M. Kay, W. J. Freeman, Bidirectional processing in the olfactory-limbic axis during olfactory behavior. *Behav. Neurosci.* **112**, 541–53 (1998).

40. I. Fukunaga, J. T. Herb, M. Kollo, E. S. Boyden, A. T. Schaefer, Independent control of gamma and theta activity by distinct interneuron networks in the olfactory bulb. *Nat. Neurosci.* **17**, 1208–1216 (2014).

41. K. Mori, H. Manabe, K. Narikiyo, N. Onisawa, Olfactory consciousness and gamma oscillation couplings across the olfactory bulb, olfactory cortex, and orbitofrontal cortex. *Front. Psychol.* **4**, 743 (2013).

42. F. H. Eeckman, W. J. Freeman, Correlations between unit firing and EEG in the rat olfactory system. *Brain Res.* **528**, 238–244 (1990).

43. W. J. Freeman, C. A. Skarda, Spatial EEG patterns, non-linear dynamics and perception: The neo-Sherringtonian view. *Brain Res.* **10**, 147–175 (1985).

44. C. M. Gray, J. E. Skinner, Centrifugal regulation of neuronal activity in the olfactory bulb of the waking rabbit as revealed by reversible cryogenic blockade. *Exp. Brain Res.* **69**, 378–386 (1988).

45. B. Iravani, A. Arshamian, K. Ohla, D. A. Wilson, J. N. Lundström, Non-invasive recording from the human olfactory bulb. *Nat. Commun.* **11**, 648 (2020).

46. B. Iravani, A. Arshamian, M. Lundqvist, L. M. Kay, D. A. Wilson, J. N. Lundström, Odor identity can be extracted from the reciprocal connectivity between olfactory bulb and piriform cortex in humans. *Neuroimage* **237**, 118130 (2021).

47. A. C. Santiago, S. J. Shammah-Lagnado, Efferent, connections of the nucleus of the lateral olfactory tract in the rat. *J Comp Neurol* **471**, 314–332 (2004).

48. H. Chae, A. Banerjee, M. Dussauze, D. F. Albeanu, Long-range functional loops in the mouse olfactory system and their roles in computing odor identity. *Neuron* **110**, 3970–3985.e7 (2022).

49. R. Garg, Q. Qiu, C. R. Yu, Basal forebrain cholinergic input mediates adaptive attention allocation to enhance olfactory discrimination. *PLoS Biol.* **23**, e3003374 (2025).

50. R. Garg, V. Kumar, J. H. Seiler, Y. Wu, M. McClain, K. Yi, C. R. Yu, Dopaminergic short axon cells integrate sensory and top–down inputs to enhance discriminative learning in the mouse olfactory bulb. *PLoS Biol.* **23**, e3003375 (2025).

51. A. M. Boyd, J. F. Sturgill, C. Poo, J. S. Isaacson, Cortical feedback control of olfactory bulb circuits. *Neuron* **76**, 1161–1174 (2012).

52. D. Restrepo, W. Doucette, J. D. Whitesell, T. S. McTavish, E. Salcedo, From the top down: Flexible reading of a fragmented odor map. *Trends Neurosci.* **32**, 525–531 (2009).

53. L. M. Kay, Two species of gamma oscillations in the olfactory bulb: Dependence on behavioral state and synaptic interactions. *J. Integr. Neurosci.* **02**, 31–44 (2003).

54. D. E. Frederick, A. Brown, E. Brim, N. Mehta, M. Vujovic, L. M. Kay, Gamma and beta oscillations define a sequence of principal modes present in odor processing. *J. Neurosci.* **36**, 7750–7767 (2016).

55. G. Zhou, G. Lane, T. Noto, G. Arabkheradmand, J. A. Gottfried, S. U. Schuele, J. M. Rosenow, J. K. Olofsson, D. A. Wilson, C. Zelano, Human olfactory-auditory integration requires phase synchrony between sensory cortices. *Nat. Commun.* **10**, 1168 (2019).

56. A. Goyal, J. Miller, S. E. Qasim, A. J. Watrous, H. Zhang, J. M. Stein, C. S. Inman, R. E. Gross, J. T. Willie, B. Lega, J. J. Lin, A. Sharan, C. Wu, M. R. Sperling, S. A. Sheth, G. M. McKhann, E. H. Smith, C. Schevon, J. Jacobs, Functionally distinct high and low theta oscillations in the human hippocampus. *Nat. Commun.* **11**, 2469 (2020).

57. B. C. Lega, J. Jacobs, M. Kahana, Human hippocampal theta oscillations and the formation of episodic memories. *Hippocampus* **22**, 748–761 (2012).

58. C. Tallon-Baudry, O. Bertrand, C. Delpuech, J. Pernier, Stimulus specificity of phase-locked and non-phase-locked 40 Hz visual responses in human. *J. Neurosci.* **16**, 4240–4249 (1996).

59. J. P. Lachaux, E. Rodriguez, J. Martinerie, F. J. Varela, Measuring phase synchrony in brain signals. *Hum. Brain Mapp.* **8**, 194–208 (1999).

60. M. X. Cohen, *Analyzing Neural Time Series Data* (The MIT Press, 2014).

61. D. E. Frederick, A. Brown, E. Brim, N. Mehta, M. Vujovic, L. M. Kay, Gamma and beta oscillations define a sequence of neurocognitive modes present in odor processing. *J. Neurosci.* **36**, 7750–7767 (2016).

62. H. Wen, Z. Liu, Separating fractal and oscillatory components in the power spectrum of neurophysiological signal. *Brain Topogr.* **29**, 13–26 (2016).

63. A. B. L. Tort, M. A. Kramer, C. Thorn, D. J. Gibson, Y. Kubota, A. M. Graybiel, N. J. Kopell, Dynamic cross-frequency couplings of local field potential oscillations in rat striatum and hippocampus during performance of a T-maze task. *Proc. Natl. Acad. Sci. U.S.A.* **105**, 20517–20522 (2008).

64. R. T. Canolty, E. Edwards, S. S. Dalal, M. Soltani, S. S. Nagarajan, H. E. Kirsch, M. S. Berger, N. M. Barbaro, R. T. Knight, High gamma power is phase-locked to theta oscillations in human neocortex. *Science* **313**, 1626–1628 (2006).

65. O. Jensen, L. L. Colgin, Cross-frequency coupling between neuronal oscillations. *Trends Cogn. Sci.* **11**, 267–269 (2007).

66. J. Harvey, D. Rinberg, Olfaction: Source separation in a single sniff. *Curr. Biol.* **31**, R1051–R1053 (2021).

67. N. Ravel, J. Pager, Respiratory patterning of the rat olfactory bulb unit activity: Nasal versus tracheal breathing. *Neurosci. Lett.* **115**, 213–218 (1990).

68. T. L. Spencer, A. Clark, J. Fonollosa, E. Virot, D. L. Hu, Sniffing speeds up chemical detection by controlling air-flows near sensors. *Nat. Commun.* **12**, 1232 (2021).

69. B. H. Bland, S. D. Oddie, Theta band oscillation and synchrony in the hippocampal formation and associated structures: The case for its role in sensorimotor integration. *Behav. Brain Res.* **127**, 119–136 (2001).

70. J. J. Chrobak, G. Buzsáki, Gamma oscillations in the entorhinal cortex of the freely behaving rat. *J. Neurosci.* **18**, 388–398 (1998).

71. J. M. Hyman, E. A. Zilli, A. M. Paley, M. E. Hasselmo, Medial prefrontal cortex cells show dynamic modulation with the hippocampal theta rhythm dependent on behavior. *Hippocampus* **15**, 739–749 (2005).

72. C. Kayser, R. A. A. Ince, S. Panzeri, Analysis of slow (theta) oscillations as a potential temporal reference frame for information coding in sensory cortices. *PLOS Comput. Biol.* **8**, e1002717 (2012).

73. P. Fries, Rhythms for cognition: Communication through coherence. *Neuron* **88**, 220–235 (2015).

74. P. Fries, A mechanism for cognitive dynamics: Neuronal communication through neuronal coherence. *Trends Cogn. Sci.* **9**, 474–480 (2005).

75. M. Schneider, A. C. Broggini, B. Dann, A. Tzanou, C. Uran, S. Sheshadri, H. Scherberger, M. Vinck, A mechanism for inter-areal coherence through communication based on connectivity and oscillatory power. *Neuron* **109**, 4050–4067.e12 (2021).

76. R. T. Canolty, R. T. Knight, The functional role of cross-frequency coupling. *Trends Cogn. Sci.* **14**, 506–515 (2010).

77. M. Stopfer, S. Bhagavan, B. H. Smith, G. Laurent, Impaired odour discrimination on desynchronization of odour-encoding neural assemblies. *Nature* **390**, 70–74 (1997).

78. K. MacLeod, A. Bäcker, G. Laurent, Who reads temporal information contained across synchronized and oscillatory spike trains? *Nature* **395**, 693–698 (1998).

79. N. Gupta, S. S. Singh, M. Stopfer, Oscillatory integration windows in neurons. *Nat. Commun.* **7**, 13808 (2016).

80. T. Womelsdorf, M. Vinck, L. S. Leung, S. Everling, Selective theta-synchronization of choice-relevant information subserves goal-directed behavior. *Front. Hum. Neurosci.* **4**, 1–13 (2010).

81. T. Womelsdorf, J.-M. Schoffelen, R. Oostenveld, W. Singer, R. Desimone, A. K. Engel, P. Fries, Modulation of neuronal interactions through neuronal synchronization. *Science* **316**, 1609–12 (2007).

82. L. M. Kay, M. Stopfer, Information processing in the olfactory systems of insects and vertebrates. *Semin. Cell Dev. Biol.* **17**, 433–442 (2006).

83. K. A. Fulton, D. Zimmerman, A. Samuel, K. Vogt, S. R. Datta, Common principles for odour coding across vertebrates and invertebrates. *Nat. Rev. Neurosci.* **25**, 453–472 (2024).

84. J. A. Villanueva, D. Restrepo, D. Ramirez-Gordillo, "Closed-loop optogenetic stimulation of the olfactory circuit at different phases of theta oscillations of the local field potential" in *Methods in Molecular Biology* (Humana Press Inc., 2025), vol. **2915**, pp. 179–187.

85. T. W. Margrie, A. T. Schaefer, Theta oscillation coupled spike latencies yield computational vigour in a mammalian sensory system. *J. Physiol.* **546**, 363–374 (2003).

86. M. Mooziri, A. Samii Moghaddam, M. A. Mirshekar, M. R. Raoufy, Olfactory bulb-medial prefrontal cortex theta synchronization is associated with anxiety. *Sci. Rep.* **14**, 12101 (2024).

87. M. Salimi, F. Tabasi, M. Nazari, S. Ghazvineh, A. Salimi, H. Jamaati, M. R. Raoufy, The olfactory bulb modulates entorhinal cortex oscillations during spatial working memory. *J. Physiol. Sci.* **71**, 21 (2021).

88. S. Rafilson, N. Gonzales Hess, T. M. Findley, M. C. Smear, Challenges in inferring breathing rhythms from olfactory bulb local field potentials. *Chem. Senses* **50**, bjaf026 (2025).

89. A. Sheriff, G. Pandolfi, V. S. Nguyen, L. M. Kay, Long-range respiratory and theta oscillation networks depend on spatial sensory context. *J. Neurosci.* **41**, 9957–9970 (2021).

90. A. G. Khan, M. Sarangi, U. S. Bhalla, Rats track odour trails accurately using a multi-layered strategy with near-optimal sampling. *Nat. Commun.* **3**, 703 (2012).

91. U. Bhalla, J. M. Bower, Multiday recordings from olfactory bulb neurons in awake freely moving rats: Spatial and temporal patterns in odorant responses. *J. Comput. Neurosci.* **4**, 221–256 (1997).

92. R. M. Carey, J. V. Verhagen, D. W. Wesson, N. Pírez, M. Wachowiak, Temporal structure of receptor neuron input to the olfactory bulb imaged in behaving rats. *J. Neurophysiol.* **101**, 1073–1088 (2009).

93. V. Nguyen Chi, C. Muller, T. Wolfenstetter, Y. Yanovsky, A. Draguhn, A. B. L. Tort, J. Brankačk, Hippocampal respiration-driven rhythm distinct from theta oscillations in awake mice. *J. Neurosci.* **36**, 162–177 (2016).

94. L. M. Kay, Theta oscillations and sensorimotor performance. *Proc. Natl. Acad. Sci. U.S.A.* **102**, 3863–3868 (2005).

95. F. Macrides, H. Eichenbaum, W. Forbes, Temporal relationship between sniffing and the limbic theta rhythm during odor discrimination reversal learning. *J. Neurosci.* **2**, 1705–1717 (1982).

96. F. Macrides, Temporal relationships between hippocampal slow waves and exploratory sniffing in hamsters. *Behav. Biol.* **14**, 295–308 (1975).

97. G. Zhou, J. K. Olofsson, M. Z. Koubeissi, G. Menelaou, J. Rosenow, S. U. Schuele, P. Xu, J. L. Voss, G. Lane, C. Zelano, Human hippocampal connectivity is stronger in olfaction than other sensory systems. *Prog. Neurobiol.* **201**, 102027 (2021).

98. H. Jiang, S. Schuele, J. Rosenow, C. Zelano, J. Parvizi, J. X. Tao, S. Wu, J. A. Gottfried, Theta oscillations rapidly convey odor-specific content in human piriform cortex. *Neuron* **94**, 207–219.e4 (2017).

99. G. N. Dikeçligil, A. I. Yang, N. Sanghani, T. Lucas, H. I. Chen, K. A. Davis, J. A. Gottfried, Odor representations from the two nostrils are temporally segregated in human piriform cortex. *Curr. Biol.* **33**, 5275–5287.e5 (2023).

100. Q. Yang, G. Zhou, T. Noto, J. W. Templer, S. U. Schuele, J. M. Rosenow, G. Lane, C. Zelano, Smell-induced gamma oscillations in human olfactory cortex are required for accurate perception of odor identity. *PLOS Biol.* **20**, e3001509 (2022).

101. G. N. Dikeçligil, J. A. Gottfried, What does the human olfactory system do, and how does it do it? *Annu. Rev. Psychol.* **75**, 155–181 (2024).

102. G. Arabkheradmand, G. Zhou, T. Noto, Q. Yang, S. U. Schuele, J. Parvizi, J. A. Gottfried, S. Wu, J. M. Rosenow, M. Z. Koubeissi, G. Lane, C. Zelano, Anticipation-induced delta phase reset improves human olfactory perception. *PLOS Biol.* **18**, e3000724 (2020).

103. A. T. Schaefer, T. W. Margrie, Spatiotemporal representations in the olfactory system. *Trends Neurosci.* **30**, 92–100 (2007).

104. A. C. Marin, A. T. Schaefer, T. Ackels, Spatial information from the odour environment in mammalian olfaction. *Cell Tissue Res.* **383**, 473–483 (2021).

105. S. L. Brown, J. Joseph, M. Stopfer, Encoding a temporally structured stimulus with a temporally structured neural representation. *Nat. Neurosci.* **8**, 1568–1576 (2005).

106. C. Li, H. Dong, K. Zhao, A balance between aerodynamic and olfactory performance during flight in Drosophila. *Nat. Commun.* **9**, 3215 (2018).

107. M. A. R. Koehl, J. R. Koseff, J. P. Crimaldi, M. G. McCay, T. Cooper, M. B. Wiley, P. A. Moore, Lobster sniffing: Antennule design and hydrodynamic filtering of information in an odor plume. *Science* **294**, 1948–1951 (2001).

108. T. Kawai, H. Abe, K.-i. Wakabayashi, Y. Oka, Calcium oscillations in the olfactory nonsensory cells of the goldfish, Carassius auratus. *Biochim. Biophys. Acta* **1790**, 1681–1688 (2009).

109. J. Yager, T.-M. Chen, M. J. Dulfano, Measurement of frequency of ciliary beats of human respiratory epithelium. *Chest* **73**, 627–633 (1978).

110. D. Rojas-Líbano, L. M. Kay, Olfactory system gamma oscillations: The physiological dissection of a cognitive neural system. *Cogn. Neurodyn.* **2**, 179–194 (2008).

111. I. Fukunaga, M. Berning, M. Kollo, A. Schmaltz, A. T. Schaefer, Two distinct channels of olfactory bulb output. *Neuron* **75**, 320–329 (2012).

112. B. L. Osinski, L. M. Kay, Granule cell excitability regulates gamma and beta oscillations in a model of the olfactory bulb dendrodendritic microcircuit. *J. Neurophysiol.* **116**, 522–539 (2016).

113. Q. Li, Y. Takeuchi, J. Wang, L. Gellért, L. Barcsai, L. K. Pedraza, A. J. Nagy, G. Kozák, S. Nakai, S. Kato, K. Kobayashi, M. Ohsawa, G. Horváth, G. Kékesi, M. L. Lőrincz, O. Devinsky, G. Buzsáki, A. Berényi, Reinstating olfactory bulb-derived limbic gamma oscillations alleviates depression-like behavioral deficits in rodents. *Neuron* **111**, 2065–2075.e5 (2023).

114. T. Soroka, A. Ravia, K. Snitz, D. Honigstein, A. Weissbrod, L. Gorodisky, T. Weiss, O. Perl, N. Sobel, Humans have nasal respiratory fingerprints. *Curr. Biol.* **35**, 3011–3021.e3 (2025).

115. V. Sagar, A. Sheriff, Q. Yang, N. Arriaga, G. Zhou, G. Lane, T. Kahnt, C. Zelano, The human brain modulates sniffs according to fine-grained perceptual features of odours. *Nat. Hum. Behav.* **10**, 137–147 (2026).

116. R. L. Doty, R. E. Frye, U. Agrawal, Internal consistency reliability of the fractionated and whole University of Pennsylvania Smell Identification Test. *Percept. Psychophys.* **45**, 381–384 (1989).

117. P. Berens, CircStat: A MATLAB toolbox for circular statistics. *J. Stat. Softw.* **31**, 1–21 (2009).

118. R. Oostenveld, P. Fries, E. Maris, J. M. Schoffelen, FieldTrip: Open source software for advanced analysis of MEG, EEG, and invasive electrophysiological data. *Comput. Intell. Neurosci.* **2011**, 156869 (2011).

119. R. T. Canolty, Spatiotemporal dynamics of word processing in the human brain. *Front. Neurosci.* **1**, 185–196 (2007).

120. V. F. Low, C. Lin, S. Su, M. Osanlouy, M. Khan, S. Safaei, G. Maso Talou, M. A. Curtis, P. Mombaerts, Visualizing the human olfactory projection and ancillary structures in a 3D reconstruction. *Commun. Biol.* **7**, 1467 (2024).

121. O. Perl, A. Ravia, M. Rubinson, A. Eisen, T. Soroka, N. Mor, L. Secundo, N. Sobel, Human non-olfactory cognition phase-locked with inhalation. *Nat. Hum. Behav.* **3**, 501–512 (2019).
